# Metabolome and transcriptome integration reveals insights into the process of delayed petal abscission in rose by STS

**DOI:** 10.3389/fpls.2022.1045270

**Published:** 2022-11-15

**Authors:** Jingjing Zhang, Yuyun Zhang, Yongmei He, Tingting Du, Duoxiu Shan, Houdong Fan, Wenyu Wang, Zhe Qin, Cuihua Xin, Haixia Pei

**Affiliations:** School of Life Science ang Technology, Inner Mongolia University of Science and Technology, Baotou, China

**Keywords:** silver thiosulfate, rose, abscission, metabolome, transcriptome

## Abstract

The abscission of plant organs plays an important role in ensuring the normal life activities. Rose is one of the most important ornamental plants, and its premature abscission of petal has seriously affected the quality and commercial value. Silver Thiosulfate (STS) is an ethylene inhibitor, which is often used preservative to delay the senescence of fresh cut flowers. To understand the regulatory mechanism of petal abscission in rose by STS, integrative analysis of the metabolome and transcriptome profiles was performed in abscission zone (AZ) tissues of rose under different treatments (MOCK, STS, ETH, STS+ETH). The results showed that STS significantly delayed the petal abscission in phenotype and reduced the activity of two enzymes (pectinase and cellulase) associated with cell wall degradation in physiological level. STS affected the contents of five metabolites (shikonin, jasmonic acid, gluconolactone, stachyose and D-Erythrose 4-phosphate), and involved changes in the expression of 39 differentially expressed genes (DEGs) associated with these five metabolites. Five DEGs (*LOC112192149*, *LOC112196726*, *LOC112189737*, *LOC112188495*, and *LOC112188936*) were probably directly associated with the biosynthesis of shikonin, jasmonic acid, and D-Erythrose 4-phosphate. Meanwhile, the effect of STS on the abscission process significantly involved in the pentose phosphate pathway and amino acid biosynthesis pathway. In addition, STS had a greater effect on the transcription factors, phytohormone related DEGs represented by auxin and ethylene, DEGs related to disease resistance and amino acid, etc. Above all, STS negatively influences petal abscission of rose, these results maybe provide a reference for subsequent studies on petal abscission of rose by STS.

## 1 Introduction

The abscission of tissues and organs is important in the normal growth and reproductive development of plants (Kim et al., 2019; Liu et al., 2022). Abscission is the process by which an organ or tissue is separated from the main body of the plant. This process is usually restricted to a localized region of cells termed an abscission zone (AZ). The abscission process comprises four stages: (1) differentiation of the AZ; (2) sensing of the abscission signal in the AZ and initiation of abscission; (3) cell separation leading to abscission of the organ or tissue; and (4) differentiation of the abscission layer and protective layer (Singh et al., 2020). Premature senescence, which includes premature abscission of plant organs such as flowers and fruits, can severely affect agricultural production (Patharkar and Walker, 2018).

Changes in endogenous phytohormone contents are a critical factor in the initiation of abscission (Kim et al., 2019). Among phytohormones, ethylene is considered to be an important regulator of abscission, and many factors involved in ethylene biosynthesis and signal transduction participate in this process (Gao et al., 2016). In Arabidopsis, the abscission of floral organs is inhibited in the ethylene-insensitive mutants *etr1-1* and *ein2* (Patterson and Bleecker, 2004). The abscission of tomato pedicels is accompanied by upregulation of the expression of *1-AMINOCYCLOPROPANE-1-CARBOXYLATE SYNTHASE 1A* (*ACS1A*), *ACS2*, *ACS6*, *ACC OXIDASE 1* (*ACO1*), *ACO5*, and *ETHYLENE RESPONSE FACTOR 52* (*ERF52*) (Meir et al., 2010; Nakano et al., 2014). In lychee, a low expression level of *LcERF2* reduces the rate of fruit abscission (Yi et al., 2021). Experimental treatment with exogenous ethylene in many studies has demonstrated that the onset of abscission is strongly associated with the presence of ethylene. For example, the application of ethephon (which is converted to ethylene in plant tissues) accelerates fruit abscission in sweet cherry and sweet orange, and treatment of rose, geranium, and tulip with exogenous ethylene leads to the early onset of flower organ abscission (John-Karuppiah and Burns, 2010; Smith and Whiting, 2010; Singh et al., 2022). In contrast, inhibitors of ethylene synthesis or action effectively delay abscission, but the effect of ethylene inhibitors on the mechanism of action of ethylene and petal abscission is uncertain.

The silver thiosulfate (STS) complex is commonly used as an inhibitor of ethylene synthesis. Application of STS extends the vase life of many cut flowers, such as lily, sunflower, and carnation, but does not significantly extend the vase life of all cut flower types (Liu et al., 2018; Kılıç et al., 2020; Krause et al., 2021). For example, treatment of gardenia cut flowers with STS has no effect on the vase life, and STS treatment of goldenseal cut flowers slightly promotes flower opening but does not extend the vase life (Çelikel et al., 2020; Kato et al., 2022). In addition, STS impacts on other physiological functions in plants. For example, STS can induce the expression of androgenic-related genes in female cannabis plants to develop male flowers (Adal et al., 2021). Ethephon prevents the bending of goldenseal stems, whereas STS promotes the bending rate of goldenseal stems (Naing et al., 2021). Under a humid environment, STS reduces the emission of carnation fragrance after harvesting, thus prolonging the fragrance of carnations (Kishimoto, 2021). Supplementation of the culture medium with STS significantly increases the number of regenerating shoots per leaf and significantly improves the *in vitro* regeneration frequency of a peach rootstock (Ricci et al., 2020). Although STS solution effectively prolongs the vase life of rose cut flowers (Son et al., 2003), the detailed mechanism by which STS delays petal abscission in rose remains unclear.

Rose is among the most economically important cut flower crops worldwide. Petal senescence is the main factor that affects the rose flower quality and petal abscission is the culmination of the senescence process (Gao et al., 2016). Most previous studies of petal abscission in rose have focused on the regulatory mechanism of endogenous phytohormones, such as auxin, ethylene and jasmonic acid. In rose, silencing of the AUX/IAA gene *RhIAA16* promotes petal abscission (Gao et al., 2016). In petal abscission of rose, the ethylene receptor genes *RhERF1* and *RhERF4* coordinate ethylene and auxin signaling by reducing the expression level of the pectin-metabolizing gene beta-GALACTOSIDASE 1 (*RhBGLA1*), thereby inhibiting abscission (Gao et al., 2019). Synergistic action of the auxin response factor *RhARF7* and the sucrose transporter protein RhSUC2 inhibits ethylene-induced petal abscission (Liang et al., 2019). Ethylene promotes petal abscission of rose and affects gene expression in the AZ (Singh et al., 2020), whereas the jasmonic acid pathway is strongly regulated during abscission, which negatively affects petal abscission in rose (Singh et al., 2022). In summary, petal abscission is a complex physiological process, and the regulatory mechanisms of multiple factors, including STS, on this process require further in-depth investigation.

In this study, we analyzed the effect of STS in four treatment solutions (distilled water, STS, ETH, and STS+ETH) on petal abscission in rose cut flowers at the phenotypic, physiological, transcriptomic, and metabolomic levels. The present study provide some results into the metabolite contents and molecular mechanism of STS-delayed petal abscission of rose flowers.

## 2 Materials and methods

### 2.1 Plant materials and treatments

The ethylene-sensitive rose cultivar *R. hybrida* cv. ‘Tineke’ was as material for the experiments in this study. The fresh rose flowers at flower opening stage 2 were harvested from a flower plantation in Baotou, Inner Mongolia Autonomous Region, China (Gao et al., 2016). The rose flowers were brought back to the laboratory immediately after harvesting, then they were cut to 25 cm in length under water and placed in deionized water until further processing. The rose flowers were inserted into four different treatment solutions (deionized water, STS, ETH, STS+ETH), the number of flowers under each treatment was 36, and the experiment was repeated three times. The solution of STS was a mixture of 158 mg/L AgNO_3_ and 924 mg/L anhydrous Na_2_S_2_O_3_, the solution of ETH was configured with 25 mg/L ethephon, and the solution of STS and ETH was a 1:1 mixture of the solutions of STS and ETH separately. Rose flowers were treated with the four solutions for 8 h, and the time point at which treatments were finished was recorded as 0 hour. Then the roses were kept on cultivating in the solution of 8-Hydroxyquinoline (8-HQ) with a concentration of 200 mg/L. Here, 8-HQ only acts as a bacterial inhibitor. Meanwhile, the 8-HQ solution was changed daily and the rose flowers were cut a little under water everyday. The rose flowers in 8-HQ were placed in a light incubator at 23 ± 1°C, 60%-80% relative humidity, and a 16/8 h (light/dark) light cycle.

### 2.2 Collection of AZ samples in rose

The sampling criterion for AZ was to cut the petal base and the petal receptacle less than 1 mm in length respectively in rose petals (Gao et al., 2016), and the sample location was shown in **Supplementary Figure 1**. The AZ samples at flower opening stage 2 were recorded as untreated samples (UT). After 8 h of treatment with different treatment solutions, the solution was replaced with 8-HQ for 24 h and 48 h. Then we obtained the AZ samples in different treatments, respectively. The samples were recorded as MOCK24h, STS24h, Eth24h, S_E24h and MOCK48h, STS48h, Eth48h, S_E48h. Meanwhile, the AZ samples were immediately frozen in liquid nitrogen and stored at -80°C for subsequent experiments.

### 2.3 Observation on abscission time of rose petal under different treatments

The abscission time of rose petal in MOCK, STS, ETH and STS+ETH treatments were observed and recorded. Photographs (Canon EOS 6D Mark II) were taken from the top angle of the rose flowers every 24 h to show the phenotype in abscission process. The observations were continued until the petals completely finished abscission or dried up on the branches.

### 2.4 Determination of pectinase and cellulase activities

The AZ samples from untreated (0 h), 8 h treated with different solutions and 48 h (8 + 48 h) in 8-HQ vase solution were selected to measure pectinase and cellulase activities in rose. Pectinase and cellulase activities were assayed using kits purchased from Nanjing Jiancheng Bioengineering Institute for pectinase (Item No. A140-1-1) and cellulase (Item No. A138).

Cellulase activity was measured according to the following steps: (1) Sample pretreatment: Weigh the samples of AZ in the single rose flower, add buffer to homogenize in ice bath, 4000 rpm, centrifuge at room temperature for 10 min, take one part of the supernatant of the homogenate for testing, and the other part of the supernatant of the homogenate in boiling water bath for 5 min (to inactivate the enzyme), then take it out and cool it to make the boiling supernatant of the homogenate; (2) Enzymatic reaction: Mix the supernatant of homogenate with buffer solution and substrate in proportion, incubate at 37°C for 30 min, take it out immediately and incubate in a boiling water bath for 15 min, take it out to cool, centrifuge at 4000 rpm for 10 min at room temperature, and prepare saccharified supernatant; (3) Color reaction: Mix the saccharified supernatant and color solution in proportion, and measure the OD value at the wave length of 550 nm. Cellulase activity (U/g tissue) = [(OD of assay tube - OD of control tube)/(OD of standard tube - OD of blank tube)) × concentration of standard) (ug/mL) × volume of reaction fluid (mL)] ÷ [(volume of sample taken (mL)/volume of extract added (mL)) × sample mass (g)] ÷ reaction time (min).

Pectinase activity was measured according to the following steps: (1) Extraction of crude enzymes extract: Weigh the samples of AZ in the single rose flower, add 5-10 times the volume of the extract buffer (mL) to homogenize in ice bath; at 4°C, 10000g, centrifuge for 10 min; take the supernatant, and place it on the ice for testing; (2) Mix the supernatant with reagent 2, and react in water bath at 50°C for 30 min. Then add reagent 3, take a boiling water bath for 5 min, cool by the ice bath to stop the reaction; 8000 g, 4°C, centrifuge for 10 min; take the supernatant, and measure the absorbance value A at the wave length of 540 nm, △A=A _measuring tube_-A _control tube_; (3) Then calculate according to the formula. Pectinase activity (U/h/g tissue) = [(∆A+0.008)/3.9642] × V_inverse total_ ÷ (× W ÷ V_sample total_) ÷ T = 2.523 × (∆A+0.008) ÷ W.

### 2.5 Metabolites extraction and analysis of UHPLC-MS/MS

At the end of 8 h of different treatments of rose, the vase solutions were uniformly changed to 8-HQ to continue vialing. The petals of ethylene-treated rose started abscission after 48 h in 8-HQ vase solution, so we made 24 h as the time point for metabolome analysis. At this moment, that did not exhibit abscission phenomenon in each treatment. Metabolomic samples were selected as UT, MOCK24h, STS24h, ETH24h, S_E24h, and six biological replicates were set. Sample preparation was as follows: tissues (100 mg) were individually grounded with liquid nitrogen and the homogenate was resuspended with prechilled 80% methanol by well vortex. The samples were incubated on ice for 5 min and then were centrifuged at 15,000 g, 4°C for 20 min. Some of supernatant was diluted to final concentration containing 53% methanol by LC-MS grade water. The samples were subsequently transferred to a fresh Eppendorf tube and then were centrifuged at 15000 g, 4°C for 20 min. Finally, the supernatant was injected into the LC-MS/MS system analysis (Want et al., 2013). UHPLC-MS/MS analyses were performed using a Vanquish UHPLC system (Thermo Fisher, Germany) coupled with an Orbitrap Q Exactive™ HF mass spectrometer (Thermo Fisher, Germany) in Novogene Co., Ltd. (Beijing, China). Samples were injected onto a Hypesil Goldcolumn (100×2.1 mm, 1.9 μm) using a 17-min linear gradient at a flow rate of 0.2 mL/min. The eluents for the positive polarity mode were eluent A (0.1% FA in Water) and eluent B (Methanol). The eluents for the negative polarity mode were eluent A (5 mM ammonium acetate, pH 9.0) and eluent B (Methanol). The solvent gradient was set as follows: 2% B, 1.5 min; 2-85% B, 3 min; 85-100% B, 10 min; 100-2% B, 10.1 min; 2% B, 12 min. Q Exactive™ HF mass spectrometer was operated in positive/negative polarity mode with spray voltage of 3.5 kV, capillary temperature of 320°C, sheath gas flow rate of 35 psi and aux gas flow rate of 10 L/min, S-lens RF level of 60, Aux gas heater temperature of 350°C.

### 2.6 Data processing and metabolite identification

The raw data files generated by UHPLC-MS/MS were processed using the Compound Discoverer 3.1 (CD3.1, Thermo Fisher) to perform peak alignment, peak picking, and quantitation for each metabolite. The main parameters were set as follows: retention time tolerance, 0.2 minutes; actual mass tolerance, 5ppm; signal intensity tolerance, 30%; signal/noise ratio, 3; and minimum intensity. After that, peak intensities were normalized to the total spectral intensity. The normalized data was used to predict the molecular formula based on additive ions, molecular ion peaks and fragment ions. And then peaks were matched with the mzCloud (https://www.mzcloud.org/), mzVault and Mass List database to obtain the accurate qualitative and relative quantitative results. Statistical analyses were performed using the statistical software R (R version R-3.4.3), Python (Python 2.7.6 version) and CentOS (CentOS release 6.6), When data were not normally distributed, it was standardized according to the formula: sample raw quantitation value/(The sum of sample metabolite quantitation value/The sum of QC1 sample metabolite quantitation value) to obtain relative peak areas. And compounds whose CVs of relative peak areas in QC samples were greater than 30% were removed, and finally the identification and relative quantification results of metabolites were obtained. The screening criteria for different comparative combinations of differential metabolites were VIP > 1.0, FC > 1.2 or FC < 0.833, p-value < 0.05.

### 2.7 Samples of transcriptome preparation, library construction and sequencing

Samples were selected as UT, MOCK24h, STS24h, ETH24h, S_E24h, and three biological replicates were set for each AZ sample. Total RNA was extracted using an RNA extraction kit (Tiangen, Beijing, Biotechnology, China) according to the manufacturer’s instructions. RNA integrity was assessed using the RNA Nano 6000 Assay Kit of the Bioanalyzer 2100 system (Agilent Technologies, CA, USA). Total RNA was used as input material for the sample preparations of RNA. Firstly, mRNA was purified from total RNA using poly-T oligo-attached magnetic beads. Fragmentation was carried out using divalent cations under elevated temperature in first strand synthesis reaction buffer (5X). First strand cDNA was synthesized using random hexamer primer and M-MuLV Reverse Transcriptase, then RNaseH was used to degrade the RNA. Second strand cDNA synthesis was subsequently performed using DNA Polymerase I and dNTP. Remaining overhangs were converted into blunt ends *via* exonuclease/polymerase activities. After adenylation of 3’ ends of DNA fragments, adaptor with hairpin loop structure was ligated to prepare for hybridization. In order to select cDNA fragments of preferentially 370~420 bp in length, the library fragments were purified with AMPure XP system (Beckman Coulter, Beverly, USA). Then PCR was performed with Phusion High-Fidelity DNA polymerase, Universal PCR primers and Index (X) primer. At last, PCR products were purified by AMPure XP system and library quality was assessed on the Agilent Bioanalyzer 2100 system. The clustering of the index-coded samples was performed on a cBot Cluster Generation System using TruSeq PE Cluster Kit v3-cBot-HS (Illumia) according to the manufacturer’s instructions. After cluster generation, the library preparations were sequenced on an Illumina Novaseq platform and 150 bp paired-end reads were generated.

### 2.8 Analysis of transcriptome data

Index of the reference genome was built using Hisat2 v2.0.5. And the paired-end clean reads were aligned to the reference genome (https://www.ncbi.nlm.nih.gov/genome/11715) using Hisat2 v2.0.5. Feature Count v1.5.0-p3 was used to count the reads numbers mapped to each gene. And then FPKM of each gene was calculated based on the length of the gene and reads count mapped to this gene. FPKM, expected number of Fragments Per Kilobase of transcript sequence per Millions base pairs sequenced. Differential expression analysis of two comparative combinations was performed using the DESeq2 R package (1.20.0). The resulting P-values were adjusted using the Benjamini and Hochberg’s approach for controlling the false discovery rate. We used *p*-value < 0.05 and |log2foldchange| > 0 as thresholds for differential gene screening for different comparative combinations. We used Cluster Profiler R package (3.4.4) to test the statistical enrichment of differential expression genes in KEGG pathways.

### 2.9 qRT-PCR validation

The samples of AZ (MOCK, STS, ETH, S_E at UT, 24h and 48h) were collected for qRT-PCR validation in rose. Reverse transcription of cDNA was performed using Evo M-MLV RT Mix Kit with gDNA Clean for qPCR (Accurate Biology, Hunan, China). QRT- PCR was performed using SYBR Green Pro HS qPCR kit (Accurate Biology, Hunan, China) and a 7500 Fast Real-Time PCR System (Applied Biosystems, Foster City, USA), using *RhUBI2* as the internal reference gene (Liang et al., 2020). The primers were designed using the NCBI online tool (https://www.ncbi.nlm.nih.gov/). The sequences of primers used in this study were shown in **Supplementary Table 1**. Three biological replicates were guaranteed for each treatment, and the data was processed using 2^-△△CT^2^ (Livak and Schmittgen, 2001).

### 2.10 Statistical analysis and image processing

ANOVA analysis of variance was performed using SPSS (version 24.0, Chicago, IL, United States), and graphs were plotted using Origin 2018 (Microcal Software, Northampton, MA). Image processing software was performed using Photoshop CC (Adobe, San Jose, California, United States).

## 3 Results

### 3.1 Effects of STS treatments on petal abscission time and physiological indicators in rose

**Figure 1:**
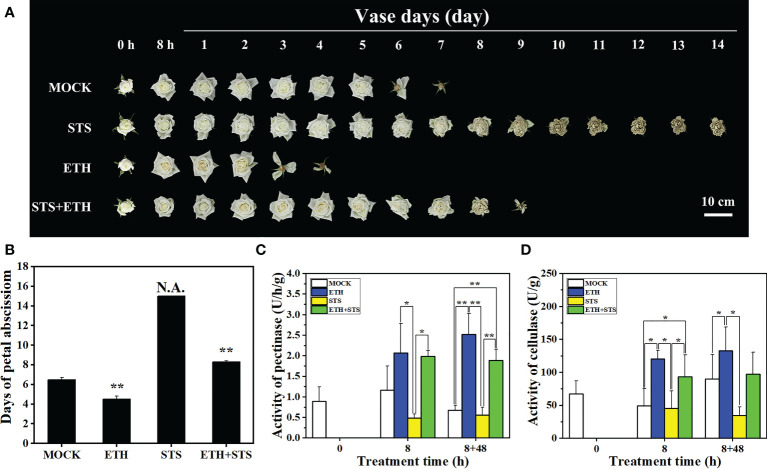
Analysis of phenotype, abscission time and enzyme activity in petal abscission process of rose. **(A, B)** Abscission phenotype and abscission time of rose petals under different treatments. 0 h, untreatment; 8 h, 8 h in treatment of different solutions; 1 to 14 days, days after treatment; N.A., no abscission. Scale bars 10 cm. **(C, D)** Changes of pectinase and cellulase activities in rose AZ. Statistical significance between different treatment and control groups was tested using Duncan’s test (*p < 0.05; **p < 0.01), biological replicates n=3.

To evaluate the potential role of STS on the process of petal abscission in rose, the phenotype, timing of petal abscission and physiological indicators were recorded and analysed. The timing of petal abscission in each treatment was ranked (from earliest to latest) as ETH > MOCK > STS + ETH > STS (**Figure 1A**). The petals of ETH-treated flowers started to abscise at 2.4 ± 0.1 d and abscised completely at 3.7 ± 0.1 d. Petal abscission in the MOCK began at 5.0 ± 0.14 d and was completed at 6.6 ± 0.1 d. In the combined STS+ETH treatment, the petals started to abscise at 7.8 ± 0.3 d and abscission was completed at 9.0 ± 0.2 d. In contrast, the petals of STS-treated flowers did not abscise, and were wilted or withered at the end of the experimental period (**Figure 1B**). Thus, ETH promoted petal abscission, whereas STS significantly inhibited the phenomenon.

The initial activation of the abscission process is usually directly associated with primary cell wall degradation, in which pectinases and cellulases are crucial promotive factors (Lashbrook and Cai, 2008). Therefore, we examined the changes in pectinase and cellulase activities in the AZ of rose petals. The comparative pectinase activity in the AZ for each treatment was ranked as ETH > STS+ETH > MOCK > STS after 8 h of treatment. This ranking remained unchanged at 48 h after the vase solution was changed 8-HQ. The cellulases activity in the treatments showed a similar pattern (**Figures 1C, D**). According to these results, primary cell wall degradation was associated with petal abscission and STS inhibited degradation of the cell wall.

### 3.2 Metabolomic analysis of rose AZ under STS treatments

To explore the changes in metabolite accumulation in the STS-treated rose, we performed UHPLC-MS/MS analysis of the AZ. The reproducibility of the metabolite assays was assessed by means of an overlap analysis using total ion chromatograms (TIC). As shown in **Supplementary Figure 2**, the peak shapes of Quality Control (QC) samples in both positive and negative ion modes were reproducible; i.e., the response intensities and retention times of the peaks were consistent. These results indicated that variation caused by instrumental errors was negligible and the mass spectrometry signals tended to be stable throughout the experiment, thus supporting the reproducibility and reliability of the metabolomics data. A total of 1120 metabolites were detected among all samples (**Supplementary Table 2**). Principal component analysis (PCA) of all metabolites detected revealed a degree of variation between samples, but little variation between samples within groups (**Supplementary Figure 3A**). The 1120 metabolites were compared against the KEGG Orthology (KO) database, which yielded 351 metabolites with annotations (**Supplementary Table 3**). These metabolites were classified into three major categories among level 1 KO pathways, namely Environmental information processing, Genetic information processing, and Metabolism. Fourteen categories were annotated among level 2 KO pathways, of which the categories annotated with a high number of metabolites were Global and overview maps (127 species), Amino acid metabolism (50 species), Biosynthesis of other secondary metabolites (46 species), Carbohydrate metabolism (29 species), and Metabolism of cofactors and vitamins (25 species). The remaining metabolites were annotated to Membrane transport, Signal transduction, Folding, sorting and degradation, Translation, Energy metabolism, Lipid metabolism, Metabolism of terpenoids and polyketides, and Nucleotide metabolism (**Supplementary Table 4**).

### 3.3 Identification of differentially accumulated metabolites

#### 3.3.1 Screening and acquisition of DAMs

**Figure 2:**
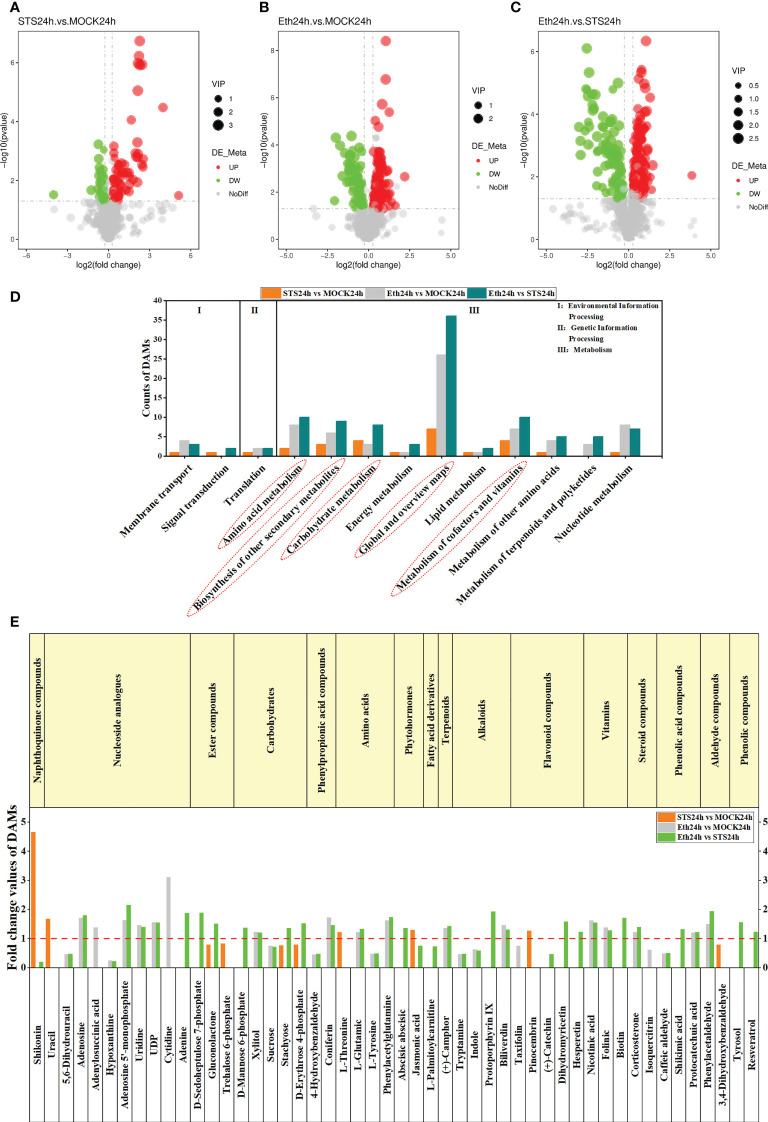
Analysis of DAMs in different comparative combinations. **(A–C)** Volcano plots of DAMs in groups STS24h vs. MOCK24h, ETH24h vs. MOCK24h and ETH24h vs. STS24h. Red represented up-regulated, green represented down-regulated. **(D)** KEGG enrichment analysis of DAMs in different comparative combinations. The content of the red dashed box was the five KEGG categories with a high number of DAMs. **(E)** Classification of the 50 DAMs and the FC values in groups STS24h vs. MOCK24h, ETH24h vs. MOCK24h and ETH24h vs. STS24h. The red dashed line was located at y-axis=1. The upper part of the dashed line indicated upward adjustment of metabolite content and the lower part indicated downward adjustment. The content of the yellow boxes indicated the different classes of metabolites.

To systematically identify metabolites that were differentially accumulated in the AZ of petals in STS-treated flowers, differentially accumulated metabolites (DAMs) of different comparative combinations were screened based on three criteria: variable importance in the projection (VIP) indicates the contribution of metabolites to the grouping; fold change (FC) is the ratio of the mean quantitative value for all biological replicates of each metabolite in the comparison group; and the *p*-value was calculated with Student’s *t*-test and indicates the degree of significance of the difference. The thresholds VIP > 1.0, FC > 1.2 or FC < 0.833, and *p*-value < 0.05 were set for screening DAMs. We identified 67 up-regulated and 40 down-regulated DAMs in the group STS24h *vs.* MOCK24h, 143 up-regulated and 72 down-regulated DAMs in the group ETH24h vs. MOCK24h, and 161 up-regulated and 92 down-regulated DAMs in the group ETH24h vs. STS24h (**Figures 2A-C**). In addition, 63 and 64 down-regulated DAMs, and 98 and 70 up-regulated DAMs were identified in the groups S_E24 h *vs.* MOCK24h and MOCK24h vs. Untreated, respectively (**Supplementary Table 5**). The DAMs in the different comparative groups were then annotated separately based on the KEGG pathway database (**Supplementary Table 6**). For the groups STS24h vs. MOCK24h, ETH24h vs. MOCK24h, and ETH24h vs. STS24h, five metabolic pathways (Global and overview maps, Amino acid metabolism, Biosynthesis of other secondary metabolites, Carbohydrate metabolism, and Metabolism of cofactors and vitamins) included significantly more DAMs than the other categories (**Figure 2D**). Similar results were observed for the groups S_E24h vs. MOCK24h and MOCK24h vs. Untreated (**Supplementary Table 6**). Therefore, we inferred that the metabolites that changed in accumulation during petal abscission in rose mainly originated from these five metabolic pathways, and that most metabolites affected by STS were also concentrated among these pathways.

#### 3.3.2 Classification of 50 DAMs from the five typical metabolic pathways

Next, we screened and classified the DAMs annotated with the aforementioned five metabolic pathways from the groups STS24h vs. MOCK24h, ETH24h vs. MOCK24h, and ETH24h vs. STS24h. Fifty metabolites were screened into 16 classes of compounds, namely, naphthoquinones (1), nucleotides (6), esters (3), carbohydrates (5), phenylpropanoids (2), amino acids (4), phytohormones (2), fatty acid derivatives (1), terpenoids (1), alkaloids (4), flavonoids (5), vitamins (3), steroids (2), phenolic acids (3), aldehydes (2), and phenolic compounds (2) (**Figure 2E**). The FC values of the 50 metabolites for the three groups are provided in **Supplementary Table 7**. Among these compounds, we observed that contents of shikonin (naphthoquinones) and jasmonic acid (phytohormones) were significantly higher in STS24h samples than in MOCK24h and ETH24h samples, and that gluconolactone (esters), stachyose (carbohydrates), and D-erythrose 4-phosphate (carbohydrates) were significantly lower in the STS24h samples than in the MOCK24h and ETH24h samples. No significant change was detected among these five metabolites in the group ETH24h vs. MOCK24h, indicating that these five metabolites changed mainly in response to STS treatment. In addition, in ETH24h samples, the contents of hypoxanthine (nucleosides), 5,6-dihydrouracil (nucleosides), uridine (nucleosides), UDP (nucleosides), sucrose (carbohydrates), 4-hydroxybenzaldehyde (phenylpropanoids), L-tyrosine (amino acids), tryptamine (alkaloids), indole (alkaloids), and caffeic aldehyde (phenolic acids) were substantially lower than those in the MOCK24h and STS24h samples; furthermore, accumulation of adenosine (nucleosides), adenosine 5′-monophosphate (nucleosides), xylitol (carbohydrates), coniferin (phenylpropanoids), L-glutamic acid (amino acids), phenylacetylglutamine (amino acids), (+)-camphor (terpenoids), biliverdin (alkaloids), nicotinic acid (vitamins), folinic acid (vitamin), corticosterone (steroidal compound), protocatechuic acid (phenolic acid), and phenylacetaldehyde (aldehyde) was much higher in the ETH24h samples than in the MOCK24h and STS24h samples. No differences were detected in the contents of the aforementioned 23 metabolites in the group STS24h vs. MOCK24h. Therefore, it was speculated that the effect of STS on petal abscission in rose may be less dependent on the production of these metabolites. In contrast, the content of the other 22 metabolites varied to different degrees in different comparisons between samples. Most of the nucleosides, amino acids, and alkaloids were affected by ethylene, whereas STS had almost no effect on these classes of metabolites (**Figure 2E**). In conclusion, these 50 metabolites may have different roles in the STS-mediated delay of petal abscission in rose.

#### 3.3.3 KEGG enrichment analysis of 50 DAMs

The analysis of DAMs in the different comparative groups indicated that Global and overview maps, Amino acid metabolism, Biosynthesis of other secondary metabolites, Carbohydrate metabolism, and Metabolism of cofactors and vitamins comprised significantly more DAMs than the other categories. Therefore, the DAMs classified to these five categories in the different comparative groups were annotated separately with KEGG pathways in accordance with the significance criterion of *p*-value < 0.05. No significantly enriched KEGG pathways were detected in the group MOCK24h vs. Untreated. The Pentose phosphate pathway and Carbon metabolism pathway were significantly enriched in the group STS24h vs. MOCK24h. Here, the Pentose phosphate pathway is the part of the Carbon metabolism. Therefore the Pentose phosphate pathway was significantly enriched, so was Carbon metabolism pathway. Pyrimidine metabolism pathway and Purine metabolism pathway were significantly enriched in the group ETH24h vs. MOCK24h. Only Arginine and proline metabolism were significantly enriched in the group S_E24h vs. MOCK24h, whereas in the group ETH24h vs. STS24h Phenylalanine, tyrosine and tryptophan biosynthesis, Pentose phosphate pathway, and Zeatin biosynthesis were significantly enriched (**Figures 5D–F** and **Supplementary Figure 4C**). Among the enriched pathways, the Pentose phosphate pathway was enriched only in the groups STS24h vs. MOCK24h, S_E24h vs. MOCK24h, and ETH24h vs. STS24h. Only the top 20 pathways ranked by *p*-value are shown in **Figures 5D-F** and **Supplementary Figure 4C**. Given that the group MOCK24h *vs.* Untreated contained no significantly enriched KEGG pathways, no KEGG bubble plot was generated. The specific KEGG enrichment results for each comparison group were shown in **Supplementary Table 8**.

### 3.4 Transcriptome analysis of rose AZ under STS treatment

#### 3.4.1 Quality assessment of transcriptome data and counting of DEGs

**Figure 3:**
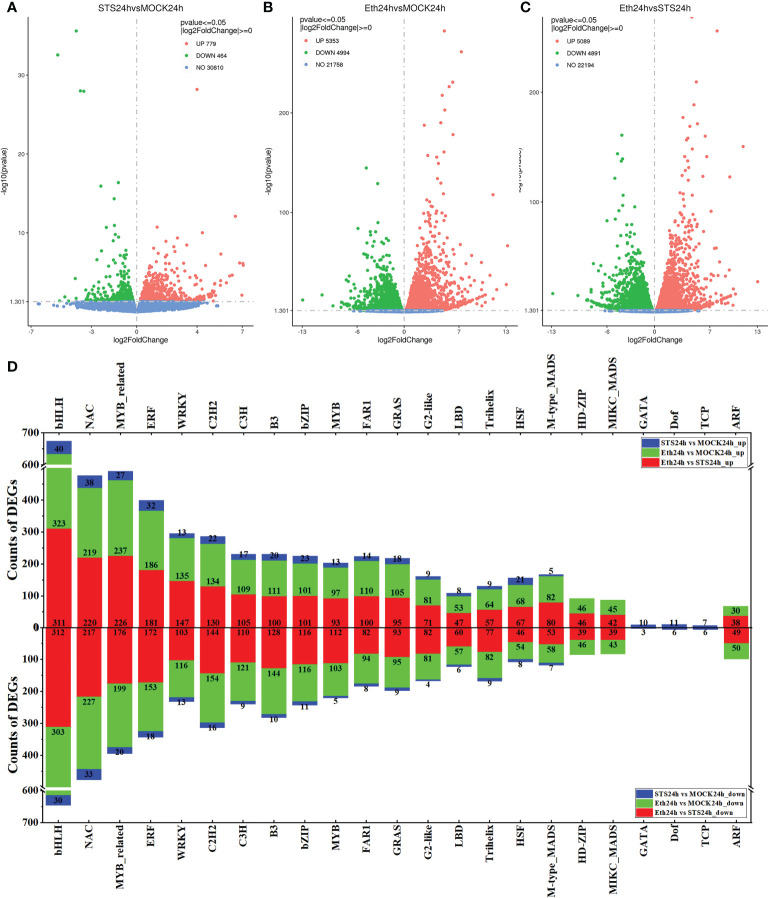
Volcano plots of DEGs and classification statistics of transcription factors. **(A–C)** Volcano plots. Red represented up-regulated, green represented down-regulated. **(D)** Classification statistics in the top 20 families of transcription factor.

The phenotypic observations, determination of pectinases and cellulases activities in the petal AZ of rose, and metabolome analysis revealed distinct differences in the STS treatment compared with the MOCK and ETH treatment groups. Therefore, to identify the genes involved in the regulation of rose petal abscission that were affected by STS, transcriptome sequencing of five groups of samples (Untreated, MOCK24h, STS24h, ETH24h, and S_E24h) was performed. In total, six G clean reads were obtained with Q20 > 97%, Q30 > 92%, and the GC content ranged between 45% and 47%. Between 85% and 88% of the clean reads were mapped to the reference genome using HISAT2 software. The data quality of each sample met the quality control criteria (**Supplementary Table 9**). On this basis, the transcriptome sequencing data were analyzed further. Differentially expressed genes (DEGs) in the different comparative groups were screened using the criteria *p*-value ≤ 0.05 and |log2 FC| ≥ 0. A total of 779 DEGs were up-regulated and 464 DEGs were down-regulated in the group STS24h vs. MOCK24h, 5353 DEGs were up-regulated and 4994 DEGs were down-regulated in the group ETH24h vs. MOCK24h, and 5089 DEGs were up-regulated and 4897 DEGs were down-regulated in the group ETH24h vs. STS24h (**Figures 3A-C** and **Supplementary Table 10**). In addition, 2587 and 1242 up-regulated DEGs and 3411 and 969 down-regulated DEGs were identified for the groups MOCK24h *vs.* Untreated and S_E24h *vs.* MOCK24h, respectively (**Supplementary Figures 5A, B**).

#### 3.4.2 Transcription factors of rose AZ affected by STS

Among the screened DEGs, the expression of many transcription factors was indicated to have changed during the petal abscission process. Therefore, we performed transcription factor (TF) categorization analysis on the screened DEGs. The DEGs in the group MOCK24h vs. Untreated included 55 TF families, those of the group STS24h vs. MOCK24h contained 50 TF families, the group ETH24h vs. MOCK24h included 57 TF families, the group S_E24h vs. MOCK24h DEGs encompassed 53 TF families, and DEGs from 56 TF families were detected in the group ETH24h *vs.* STS24h (**Supplementary Table 11**). To explore the TF families strongly influenced by STS during petal abscission, we compared the 20 top-ranked TF families by number of DEGs in each comparative group. In STS24h vs. MOCK24h, Top 20 TF families comprised bHLH (40 up-regulated, 30 down-regulated), NAC (38 up-regulated, 33 down-regulated), MYB_related (27 up-regulated, 20 down-regulated), ERF (32 up-regulated, 18 down-regulated), WRKY (13 up-regulated, 13 down-regulated), C2H2 (22 up-regulated, 16 down-regulated), C3H3 (17 up-regulated, nine down-regulated), B3 (20 up-regulated, 10 down-regulated), bZIP (23 up-regulated, 11 down-regulated), MYB (13 up-regulated, five down-regulated), FAR1 (14 up-regulated, eight down-regulated), GRAS (18 up-regulated, nine down-regulated), G2-like (nine up-regulated, four down-regulated), LBD (eight up-regulated, six down-regulated), Trihelix (nine up-regulated, nine down-regulated), HSF (21 up-regulated, eight down-regulated), M-type_MADS (five up-regulated, seven down-regulated), GATA (10 up-regulated, three down-regulated), Dof (11 up-regulated, six down-regulated), and TCP (seven up-regulated, six down-regulated) (**Figure 3D**). The number of DEGs associated with TFs in group Eth24h *vs.* STS24h was quite large. For example, the number of bHLH transcription factors reached 623, NAC transcription factors reached 437, MYB_related transcription factors reached 402, ERF transcription factors reached 353, C2H2 transcription factors reached 274 and WRKY-related transcription factors reached 250, while the number of these transcription family-related DEGs was still relatively large in group Eth24h vs. MOCK24h. Interestingly, we found significantly fewer bHLH (130), NAC (109), MYB_related (89), ERF (74), C2H2 (64) and WRKY (41) related DEGs in group S_E24h vs. MOCK24h than in group Eth24h vs. MOCK24h.This showed that when ethylene and STS were applied at the same time, STS inhibited the effect of ethylene. In summary, the effect of STS on the process of petal abscission in rose involved changes in the expression of a large number of transcription factors and STS could inhibit the regulation of a large number of transcription factors by ethylene.

#### 3.4.3 DEGs related to phytohormone biosynthesis and signal transduction in STS-treated rose AZ

**Figure 4:**
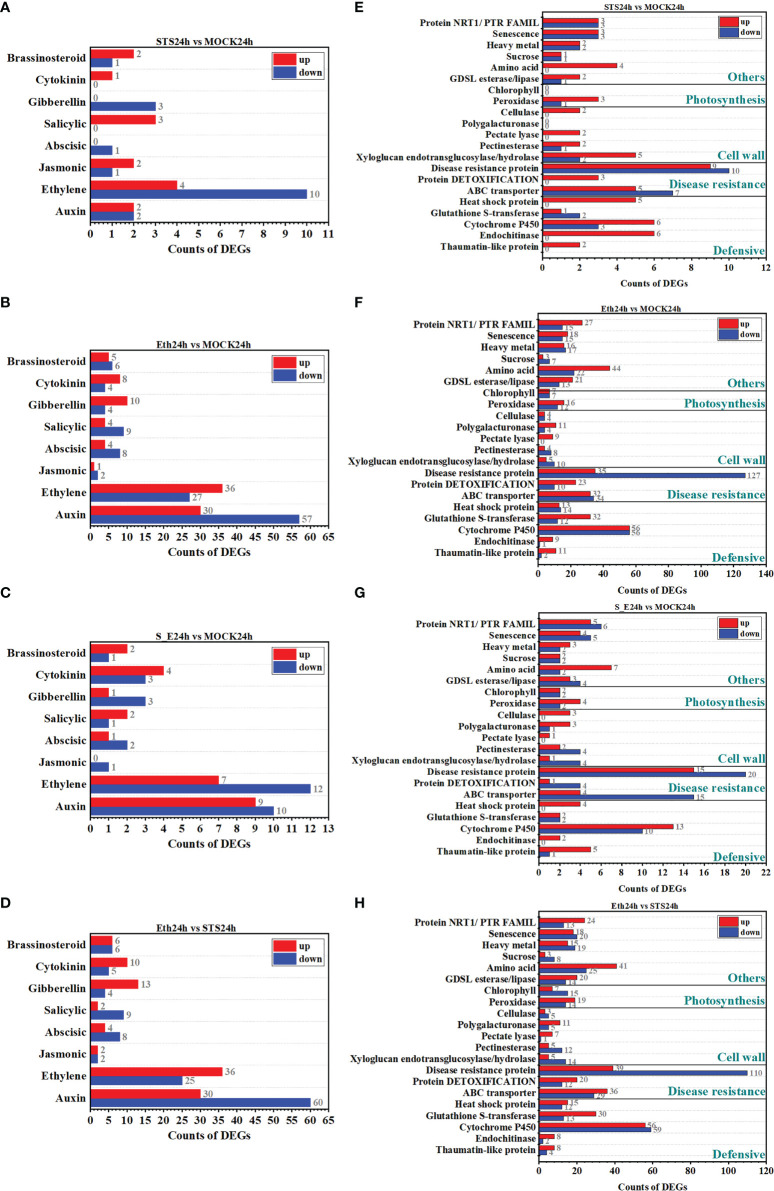
Classification of DEGs. **(A**–**D)** Classification of DEGs related to phytohormone. **(E–H)** Classification of different functionally relevant DEGs.

Phytohormone are vital regulators of plant growth and development and play an important role in organ abscission (Patharkar and Walker, 2018). To investigate whether the regulation of petal abscission by STS involved the roles of phytohormone, we analyzed the screened DEGs associated with auxin, ethylene, jasmonic acid, abscisic acid (ABA), salicylic acid, gibberellin, cytokinin, and brassinosteroids. The results showed that DEGs associated with the eight types of phytohormones were detected in almost all treatment (**Figures 4A-D** and **Supplementary Figure 5C**). Significantly more ethylene- and auxin-related DEGs were detected in the groups STS24h vs. MOCK24h, ETH24h vs. MOCK24h, and ETH24h vs. STS24h than genes associated with other phytohormones. In the group STS24h vs. MOCK24h, four auxin-related DEGs (2 up-regulated and 2 down-regulated) and 14 ethylene-related DEGs (4 up-regulated and 10 down-regulated) were detected. In the group ETH24h vs. MOCK24h, 87 auxin-related DEGs (30 up-regulated and 57 down-regulated) and 63 ethylene-related DEGs (36 up-regulated and 27 down-regulated) were identified. Among the DEGs for the group ETH24h vs. STS24h, 90 were auxin-related (30 up-regulated and 60 down-regulated) and 61 were ethylene-related (36 up-regulated and 25 down-regulated). These results indicated that the abscission process involved changes in multiple phytohormone-related genes, but the effects of STS and ethylene on phytohormones during petal abscission in rose were mainly focused on ethylene- and auxin-related genes.

#### 3.4.4 Analysis of other DEGs possibly involved rose petal abscission in STS treatment

Organ abscission is a complex physiological process involving changes in the expression of many genes with diverse functions. Therefore, we classified the DEGs into gene classes that may be involved in the petal abscission process (Singh et al., 2020). The genes were classified into 21 categories, comprising defense-related genes (thaumatin-like protein, endochitinase, cytochrome P450, glutathione *S*-transferase, and heat shock protein), disease-resistance-related genes (ABC transporter, protein detoxification, and disease resistance protein), cell wall-related genes (xyloglucan endotransglucosylase/hydrolase, pectinesterase, pectate lyase, polygalacturonase, and cellulase), photosynthesis-related genes (peroxidase and chlorophyll), and other categories of genes (GDSL esterase/lipase, amino acid, sucrose, heavy metal, senescence, and NRT1/PTR family). The numbers of DEGs in the cytochrome P450, ABC transporter, and disease resistance protein-related categories were significantly greater in the five comparative groups (STS24h vs. MOCK24h, Eth24h vs. STS24h, Eth24h vs. MOCK24h, S_E24h vs. MOCK24h and MOCK24h vs. Untreated) than for the other gene categories (**Figures 4E-H** and **Supplementary Figure 5D**). Approximately two-thirds of the disease resistance protein-related DEGs were down-regulated in the groups ETH24h vs. MOCK24h and ETH24h vs. STS24h, indicating that ethylene had a greater inhibitory effect on the expression of this class of genes. The number of photosynthesis-related DEGs were 37 in the group MOCK24h vs. Untreated and 55 in the group ETH24h vs. STS24h, whereas the number of photosynthesis-related DEGs was lower in the group STS24h vs. MOCK24h. Unlike other comparative groups, only a small number of amino acid-related DEGs were detected in STS24h and S_E24h samples compared with MOCK24h, respectively. However, a greater number of amino acid-related DEGs were observed in the groups ETH24h vs. MOCK24h and ETH24h vs. STS24h. Therefore, ethylene might strongly affect the expression of amino acid-related genes during abscission, which was distinct from the effect of STS. In this study, cell wall-associated DEGs were detected in all comparative combinations. Combined with the estimations of pectinases and cellulases activities, this result suggested a strong link existed between cell wall degradation and STS-affected abscission in rose.

#### 3.4.5 KEGG enrichment analysis of DEGs

**Figure 5:**
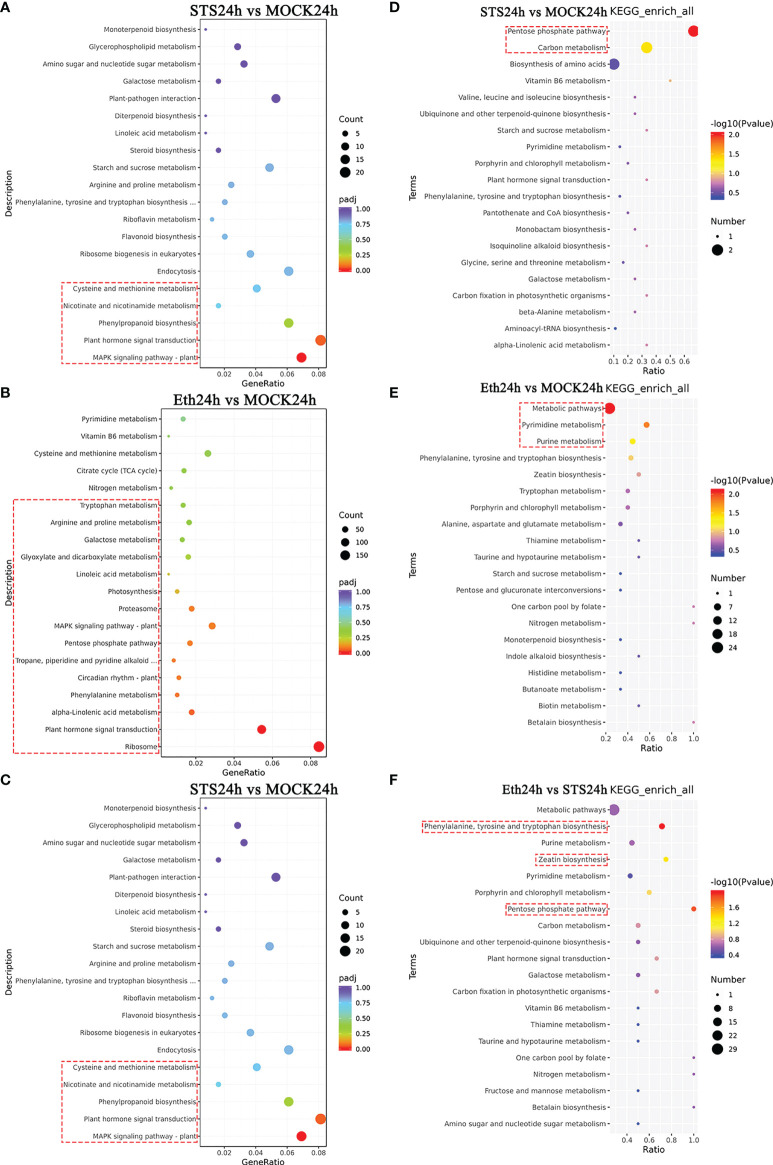
KEGG enrichment analysis of DAMs and DEGs. **(A–C)** KEGG enrichment analysis of 50 DAMs. **(D–F)** KEGG enrichment analysis of DEGs. The red dashed boxes indicated the KEGG pathways that were significantly enriched (p-value < 0.05).

Based on the screening results of DEGs, we conducted a KEGG pathway enrichment analysis in the five comparative groups. The 20 highest-ranked pathways by *p*-value were identified (**Figures 5A-C** and **Supplementary Figures 4A, B**). The specific KEGG enrichment results were shown in **Supplementary Table 12**. The pathways significantly enriched in group STS24h vs. MOCK24h were MAPK signaling pathway-plant, Plant hormone signal transduction, Phenylpropanoid biosynthesis, Nicotinate and nicotinamide metabolism, and Cysteine and methionine metabolism. Among them, MAPK signaling pathway-plant and Plant hormone signal transduction all belonged to the signal transduction class of KEGG pathways. MAPK signaling pathway-plant was also significantly enriched in the other four comparative groups, and the Plant hormone signal transduction pathway was also significantly enriched in the other three groups except for MOCK24h vs. Untreated. In addition, Phenylpropanoid biosynthesis was significantly enriched in group STS24h vs. MOCK24h; Nicotinate and nicotinamide metabolism was also significantly enriched in group S_E24h vs. MOCK24h; Cysteine and methionine metabolism was also significantly enriched in group Eth24h vs. STS24h. Therefore, in the process of petal abscission in rose, MAPK signaling pathway-plant, Plant hormone signal transduction, and Cysteine and methionine metabolism were associated with both STS and ethylene; while Phenylpropanoid biosynthesis and Nicotinate and nicotinamide metabolism pathways more depended on STS.

There were 15, 18, 9, and 19 significantly enriched KEGG pathways in the groups Eth24h vs. MOCK24h, Eth24h vs. STS24h, S_E24h vs. MOCK24h, and MOCK24h vs. Untreated, separately (**Supplementary Table 12**). According to the functional classification results of DEGs, a large number of photosynthesis-related DEGs were found in the four groups except for STS24h vs. MOCK24h. In the group MOCK24h vs. Untreated, the energy metabolism classes Photosynthesis, Photosynthesis - antenna proteins, and Carbon fixation in photosynthetic organisms were significantly enriched and all were associated directly with photosynthesis. In the groups ETH24h vs. MOCK24h (Photosynthesis), S_E24h vs. MOCK24h (Photosynthesis, Carbon fixation in photosynthetic organisms, and Nitrogen metabolism), and Eth24h vs. STS24h (Nitrogen metabolism), the specified KEGG pathways were directly associated with photosynthesis. Interestingly, no significant enrichment of photosynthesis-related KEGG pathways was detected in the group STS24h vs. MOCK24h. Based on these results, we inferred that the abscission of rose petals involved alteration of photosynthesis, and that the effect of STS was relatively less dependent on pathways involved in photosynthetic changes, which was consistent with the preceding results in KEGG enrichment analysis of DAMs.

### 3.5 Verification of DEGs from transcriptome data by qRT-PCR

**Figure 6:**
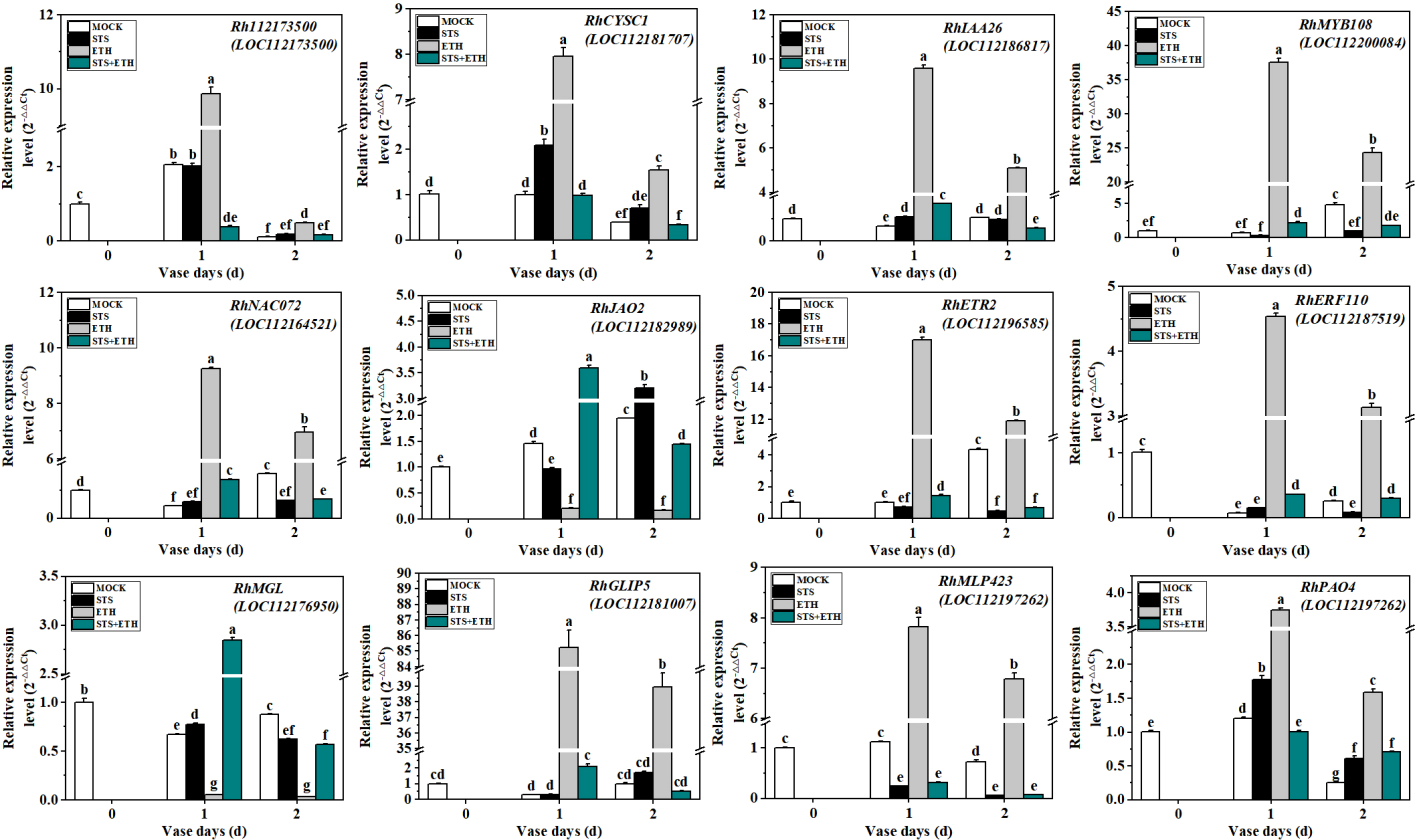
Validation of 12 DEGs by qRT-PCR. The experimental method used was 2^-△△CT^2^. *RhUBI2* was used as the internal reference gene. Different letters represent significant differences based on one-way ANOVA and Duncan's tests (p < 0.05).

To verify the reliability of the transcriptome data, we randomly selected 12 DEGs for qRT-PCR analysis based on the results of the transcriptomic and metabolomic analyses. The selected DEGs comprised *Rh112173500* (Gene ID: 112173500, F-box protein), *RhCYSC1* (Gene ID: 112181707, associated with carbon metabolism and amino acid biosynthesis), *RhIAA26* (Gene ID: 112186817, auxin response protein), *RhMYB108* (Gene ID: 112200084, MYB TF), *RhNAC072* (Gene ID: 112164521, NAC TF), *RhJAO2* (Gene ID: 112182989, associated with jasmonate synthesis), *RhETR2* (Gene ID: 112196585, ethylene receptor 2), *RhERF110* (Gene ID: 112187519, ethylene-responsive TF 110), *RhMGL* (Gene ID: 112176950, methionine gamma-lyase), *RhGLIP5* (Gene ID: 112181007, GDSL lipase), *RhMLP423* (Gene ID: 112197262, pathogenesis-associated protein), and *RhPAO4* (Gene ID: 112191920, bHLH TF). The changes in expression of these 12 DEGs in the AZ of rose petals during abscission under the different treatments showed good agreement between the transcriptome sequencing data and the qRT-PCR results (**Figure 6** and **Supplementary Table 13**). All these genes showed different changes in expression trends under the different treatments, indicating that their expression during petal abscission in rose was differentially affected by STS and ethylene.

### 3.6 Comparison between KEGG enrichment analysis of DEGs and DAMs

#### 3.6.1 Highly consistent result in KEGG enrichment analysis of DEGs and DAMs

To determine whether DEGs and DAMs in the different comparative groups were correlated in KEGG pathways, the KEGG pathways of DEGs were compared with those pathways involved in the five major classes of DAMs (Global and overview maps, Amino acid metabolism, Biosynthesis of other secondary metabolites, Carbohydrate metabolism, and Metabolism of cofactors and vitamins, **Supplementary Table 14**). All KEGG pathways of DEGs were consistent with at least half of the KEGG pathways involved in the five major classes of metabolites. These results indicated that the KEGG pathway enrichment among transcriptomic DEGs was similar to the KEGG pathway annotation of metabolomic DAMs.

#### 3.6.2 The typical KEGG pathway affected by STS

**Figure 7:**
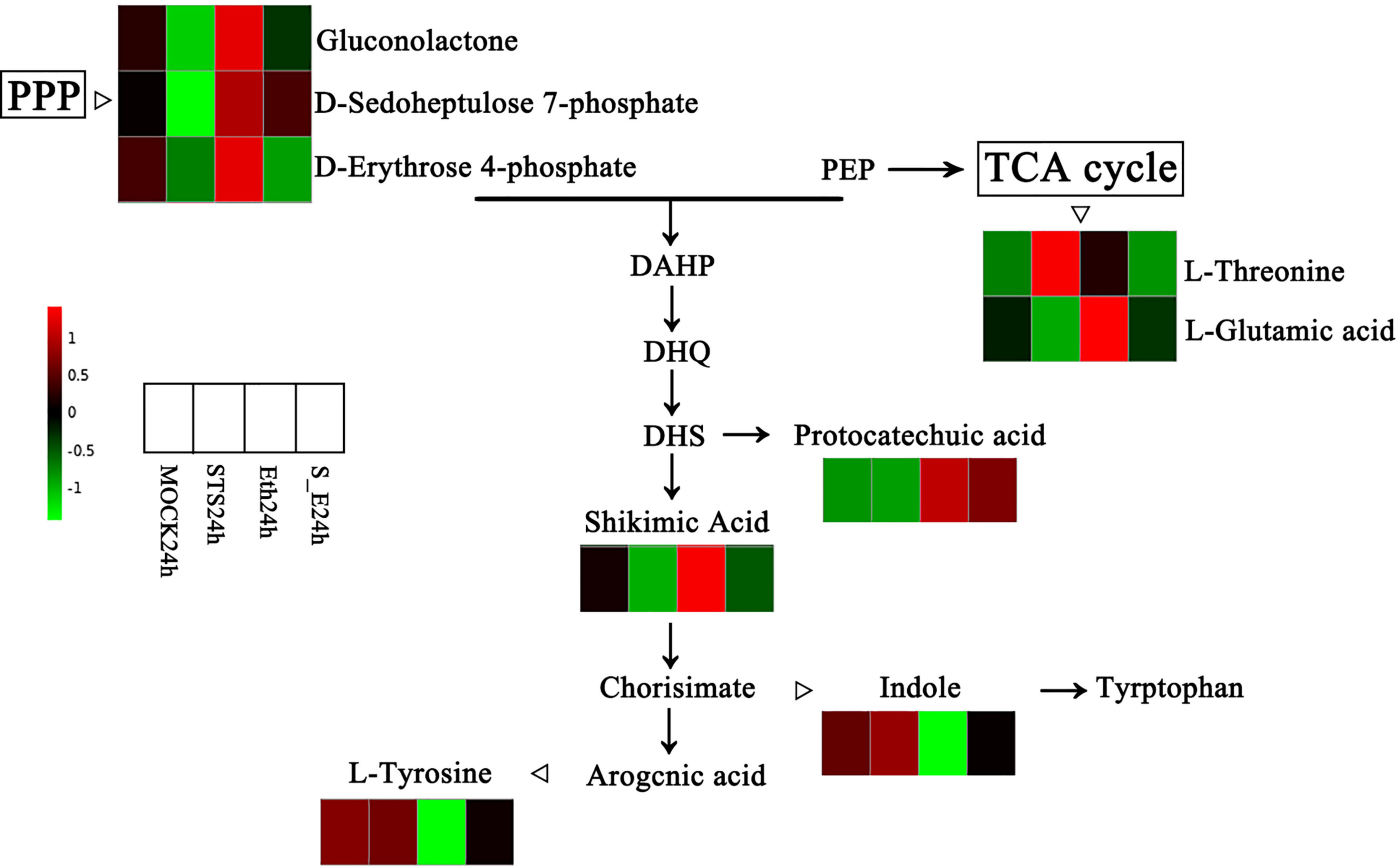
Changes of DAMs in the pentose phosphate pathway and amino acid biosynthesis. PPP, Pentose phosphate pathway; PEP, phosphoenolpyruvate; DAHP, 3-deoxy-α-arabinoheptulosonate-7-phosphate; DHQ, 3-dehydroquinate; DHS, 3-dehydroshikimate.

In order to verify the typical KEGG pathway affected by STS, we further analysed KEGG pathways of DEGs and DAMs. We found that the Pentose phosphate pathway was significantly enriched in both of DEGs and DAMs KEGG pathways in group Eth24h vs. STS24h, and gluconolactone, D-Sedoheptulose 7-phosphate and D-Erythrose 4-phosphate enriched to this pathway were differentially changed. In the group ETH24h vs. MOCK24h, Phenylalanine metabolism, Arginine and proline metabolism, and Tryptophan metabolism were associated with Amino acid metabolism, and the Pentose phosphate pathway, Glyoxylate and dicarboxylate metabolism, and Galactose metabolism were associated with Carbohydrate metabolism. The number of these significantly enriched KEGG pathways accounted for almost 85% of the KEGG pathways annotated for the five classes of DAMs for this group. Therefore, we hypothesized that amino acid metabolism and carbohydrate metabolism played a crucial role in the effect of ethylene on the abscission process in rose. Interestingly, amino acid biosynthesis involves the generation of D-Erythrose 4-phosphate, an intermediate product of the Pentose phosphate pathway (PPP). Based on this, we observed the variation patterns of DAMs in the Pentose phosphate pathway and amino acid biosynthesis (**Figure 7**). Among them, gluconolactone, D-Sedoheptulose 7-phosphate, D-Erythrose 4-phosphate, L-Glutamic acid, protocatechuic acid and shikimic acid were negatively related to STS and positively related to ethylene; L-Threonine, Indole and L-Tyrosine were positively related to STS and negatively related to ethylene. In conclusion, STS-delayed abscission of rose petal involved the pathways of the pentose phosphate pathway and amino acid biosynthesis.

### 3.7 Integral analysis of STS-affected DAMs and directly associated genes

**Figure 8:**
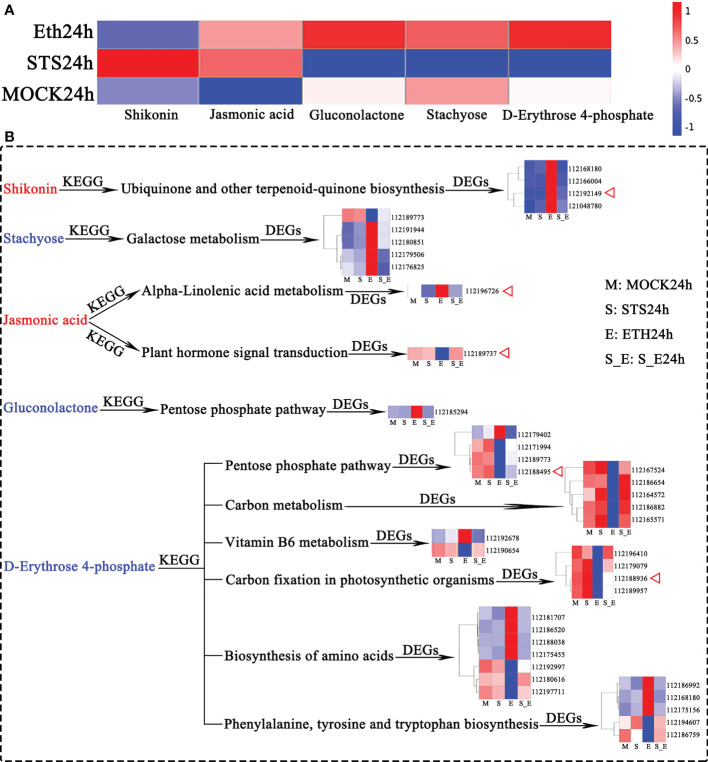
Analysis of DAMs and associated DEGs affected by STS. **(A)** Accumulation patterns of five metabolites in different samples. **(B)** DEGs associated with 5 DAMs. Red letter represented up-regulated metabolites affected by STS, blue letter represented down-regulated metabolites affected by STS. DEGs are shown for gene IDs.

The preceding identification of DAMs revealed that changes in the contents of shikonin (naphthoquinones), jasmonic acid (phytohormones), gluconolactone (esters), stachyose (carbohydrates), and D-erythrose 4-phosphate (carbohydrates) in AZ were strongly influenced by STS, but not significantly influenced by Ethylene (**Figure 2D**); therefore, we speculated that these five metabolites played an important role in the delayed petal abscission of rose by STS. Among them, shikonin and jasmonic acid were positively correlated with STS, gluconolactone, stachyose and D-Erythrose 4-phosphate were negatively correlated with STS (**Figure 8A**).

The production and changes in contents of metabolites are determined by the expression of the relevant genes in the corresponding metabolic pathways. The relevant KEGG pathways corresponding to these five metabolites were compared with the KEGG pathways for transcriptomic DEGs to identify the associated DEGs (**Figure 8B** and **Supplementary Table 15**). Shikonin is synthesized in the Ubiquinone and other terpenoid-quinone biosynthesis metabolic pathway, and four DEGs associated with shikonin were detected in this pathway (112168180, tyrosine transaminase 2; 112166004, NAD(P)H dehydrogenase (quinone); 112192149, 4-coumarate-CoA ligase 6; and 1121048780, 4-hydroxyphenylpyruvate dioxygenase). Stachyose is involved in Galactose metabolism and five associated DEGs were detected (112189773, phosphofructokinase 3; 112191944, cottonseed glycosylase 2; 112180851, galactose variable cyclase; 112179506, galactosidase; and 112176825, acidic β-fructosidase). Only one DEG (112185294, glucokinase) associated with gluconolactone in the Pentose phase pathway was detected. Jasmonic acid is associated with alpha-Linolenic acid metabolism and Plant hormone signal transduction; two associated DEGs (112196726, lipoxygenase 6; and 112189737, protein 1 containing the ZIM structural domain of jasmonic acid) were identified. D-Erythrose 4-phosphate is involved in six KEGG pathways. (1) Four DEGs were identified in the Pentose phosphate pathway (112179402, ribulose phosphate pyrophosphate kinase 1; 112171994, fructose diphosphate aldolase 2; 112189773, phosphofructokinase 3; and 112188495, glyceraldehyde-3-phosphate dehydrogenase); (2) Five DEGs were identified in Carbon metabolism (112167524, (*S*)-2-hydroxy-acid oxidase GLO1; 112186654, formate dehydrogenase; 112164572, serine-glyoxylate transaminase; 112186882, formylamine-like enzyme; and 112165571, (*S*)-2-hydroxy-acid oxidase GLO1); (3) Two DEGs (112192678, pyridoxal 5′-phosphate synthase subunit PDX1; and 112190654, inorganic pyrophosphatase 1) were identified in Vitamin B6 metabolism; (4) Four DEGs associated with Carbon fixation in photosynthetic organisms were identified (112196410, ribulose diphosphate carboxylase; 112179079, phosphokinase; 112188936, glyceraldehyde-3-phosphate dehydrogenase GAPA2; and 112189957, ribulose diphosphate carboxylase); (5) Seven DEGs involved in Biosynthesis of amino acids were detected (112181707, L-3-cyanoalanine synthase 2; 112186520, acetyl lactate synthase; 112188038, acetyl ornithine aminotransferase; 112175455, acetyl lactate synthase 2; 112192997, L-threonine aldolase 1; 112180616, glutamine synthetase cytoplasmic isozyme 2; and 112197711, glutamine synthetase leaf isozyme); (6) Five DEGs (112186992, 3-dehydroquinic acid dehydratase/manganate dehydrogenase; 112168180, tyrosine transaminase 2; 112175156, phospho-2-dehydro-3-deoxyheptulose aldolase 1; 112194607, tryptophan synthase; and 112186759, phospho-2-dehydro-3-deoxyheptulose aldolase 2) involved in Phenylalanine, tyrosine, and tryptophan biosynthesis were identified. The expression of these 39 genes showed opposite responses in the STS- and ETH-treated samples. A repressive effect of STS on ethylene was observed in the S_E samples. Significantly, the genes 112192149 (4-coumarate-CoA ligase 6), 112196726 (lipoxygenase 6), 112189737 (jasmonate ZIM domain-containing protein 1), and two glyceraldehyde-3-phosphate dehydrogenase genes (112188495 and 112188936) were key factors associated with the biosynthesis of shikonin, jasmonic acid, and D-erythrose 4-phosphate.

In summary, the changes in contents of the five metabolites were strongly associated with changes in the expression of 39 genes, which including five crucial factors related to the biosynthesis of the three metabolites. These genes may play important roles in STS-mediated inhibition of petal abscission in rose.

## 4 Discussion

The abscission of tissues or organs is a normal developmental phenomenon in plants. However, premature abscission of flowers and fruits severely affects the economic value of a plant. Previous studies have demonstrated that ethylene promotes petal abscission and affects gene expression in the AZ (Singh et al., 2020). Inhibitors of ethylene synthesis or action are effective in mitigating the negative effects of ethylene on plant organ abscission. Therefore, we delayed petal abscission by treating cut flowers of the ethylene-sensitive rose ‘Tineke’ with the ethylene inhibitor STS. The changes in the petal abscission process were compared under the MOCK, STS, ETH, and S_E24h treatments. At the physiological level, we determined changes in the activities of the cell wall-related enzymes pectinase and cellulase in the AZ. In addition, metabonomic and transcriptomic analyses were conducted to explore the changes in metabolite accumulation and the underlying molecular mechanisms in the AZ during STS-affected abscission of rose petal.

### 4.1 Effect of STS on the cell wall in rose AZ

Degradation of the primary cell wall is a major cause of plant organ abscission, and is promoted by pectinase and cellulase activity (Lashbrook and Cai, 2008). In the present study, the activities of pectinase and cellulase in the AZ under STS treatment were significantly lower than those of the MOCK and ETH-treated groups (**Figures 1C, D**). These results suggested that STS inhibited degradation of cell walls in the AZ during petal abscission and the effect was in contrast to that of ethylene. Transcriptomic analysis of DEGs revealed that, compared with the MOCK, STS affected the expression of 14 cell wall-related genes, including xyloglucan endotransglucosylase/hydrolase, pectinesterase, pectate lyase, and cellulase. Many cell wall-related genes were also affected by ethylene treatment. For example, the expression of 59 cell wall-related genes was changed in the group ETH24h vs. MOCK24h. In addition, the expression of 68 cell wall-related DEGs was changed in the group ETH24h vs. STS24h, whereas only 19 genes were differentially expressed in the group S_E vs. MOCK24h (**Figures 4E-H**). Interestingly, accumulation of gluconolactone, a metabolite associated with the cell wall, was affected by STS. Gluconolactone is able to inhibit cellulase activity in the Gramineae (Ma et al., 2022). However, in the current study, the gluconolactone content was significantly reduced under STS treatment compared with that of the MOCK and ETH treatments. These results suggested that STS affected the cell walls in the AZ during petal abscission of rose and, as an ethylene inhibitor, STS effectively alleviated the effect of ethylene on the cell wall.

### 4.2 Effects of STS on gene expression in rose AZ

Transcription factors play an important role in plant development and stress response (Niu and Fu, 2022). In this study, the DEGs affected by STS in the AZ of rose petals included a large number of TFs. The bHLH, NAC, MYB_related, ERF, WRKY, C2H2, C3H, B3, bZIP, MYB, FAR1, GRAS, G2-like, LBD, Trihelix, HSF, and M-type_MADS transcription factor families were found in the TFs with top 20 number of DEGs in each comparative combination (**Figure 3D** and **Supplementary Table 11**). In addition, the number of bHLH transcription factor family-associated DEGs was significantly more than other TFs in each comparison combination, and the number of bHLH family and NAC family DEGs was also the largest in group STS24h vs. MOCK24h. In the previous study, changes in the expression of some TFs (HSF, ARF, MYB, NAC, GRAS, SBP) were detected during rose petal abscission in response to ethylene treatment (Singh et al., 2020). And the petal abscission is characterized by complex transcriptional reprogramming, in which TF family members, such as Zinc finger, WRKY, ERF, and AUX/IAA, were differentially expressed in rose (Gao et al., 2016). The TF families HSF, MYB, NAC, GRAS, WRKY and ERF mentioned in above-mentioned study could be found in the top 20 TF families of all comparative combinations of DEGs. Besides of them, SBP TF family also present in other comparative combinations, although not in the top 20. In addition, a certain number of ARF-related TFs were present in each comparison group, 47 (17 up-regulated, 30 down-regulated) in group MOCK24h vs. Untreated; four (two up-regulated, two down-regulated) in group STS24h vs. MOCK24h; 80 (30 up-regulated, 50 down-regulated) in group ETH24h vs. MOCK24h, 21 (9 up-regulated, 12 down-regulated) in group S_E24h vs. MOCK24h, and 87 (38 up-regulated, 49 down-regulated) in group ETH24h *vs.* STS24h (**Supplementary Table 11**). Thus, the abscission of rose petals involves changes in a large number of transcription factors, and so was the role of STS in regulating the transcript levels of DEGs during abscission of rose petal.

Transcriptome sequencing results indicated that ethylene and auxin play a central role in the process of petal abscission in rose (Gao et al., 2016). Regarding phytohormone-related genes, STS and ethylene affected the expression of eight types of phytohormone-related genes, but predominantly on auxin- and ethylene-related DEGs (**Figures 4A-D**, **Supplementary Figure 5C**), and this further confirmed the previous research results (Gao et al., 2016). There were fewer auxin-related DEGs (19) and ethylene-related DEGs (19) in group S_E24h *vs.* MOCK24h than in group ETH24h vs. MOCK24h (63 auxin-related DEGs and 87 ethylene-related DEGs), suggesting that STS may mediate changes in auxin and ethylene-related gene expression through inhibition of ethylene function (**Figures 4B, C**). Meanwhile, in the results of metabolite content change analysis, we found that STS affected the content of jasmonic acid, and the expression of jasmonic acid-related genes also shew different expressions.

Among the functional classification of DEGs, the number of disease-resistant protein-associated DEGs was the most significant among all comparative combinations. There were 19 (9 up-regulated, 10 down-regulated), 162 (35 up-regulated, 127 down-regulated), and 35 (15 up-regulated, 20 down-regulated) regulated disease-resistant protein-like DEGs in groups STS24h vs. MOCK24h, ETH24h vs. MOCK24h, and S_E24h vs. MOCK24h, respectively (**Figures 4E-G**). In summary, the process of petal abscission in rose involved a lot of changes in the expression of genes related to plant disease resistance, and STS may be able to mitigate the negative effects of ethylene on plant disease resistance to some extent during petal abscission.

### 4.3 Expression analysis of DEGs by qRT-PCR in rose AZ

Combining the present results of transcriptomic and metabolomic analyses revealed that STS had significant effects on TFs, phytohormones, disease resistance, and amino acid anabolism and so on during abscission. Therefore, we randomly selected 12 DEGs that showed strong changes in expression for further analysis (**Figure 6**).

First, we screened one F-box family member *Rh112173500* and one NAC-related gene *RhNAC072*. *Rh112173500* expression was down-regulated by STS and up-regulated by ethylene. F-box family proteins play an important role in the abscission of plant organs, but their function in the onset of abscission is unclear. *NAC072* is associated with ABA-regulated senescence mechanisms in Arabidopsis. Members of the NAC TF family have been well studied in rose, but little is known about the mechanisms involved in their regulation of petal abscission (Garapati et al., 2015; Li et al., 2016). In the present study, the expression of *RhNAC072* was inhibited by STS and promoted by ethylene. Second, the expression of *RhCYSC1*, a gene associated with amino acid biosynthesis and encoding a cysteine synthase, was up-regulated under both STS and ethylene treatment. Cysteine synthase is a crucial enzyme for L-cysteine synthesis, but L-cysteine has no effect on root abscission in the water fern *Azolla pinnata* (Yamasaki et al., 2019). The onset of the abscission process is often accompanied by changes in the cell membranes, and disruption of AZ cell membranes in the AZ is associated with lipase (Kucko et al., 2022). *RhGLIP5*, a lipase-related gene, may be associated with gibberellin (GA_3_)-induced seed dormancy in *Leymus chinensis* (Li et al., 2021). In the AZ of rose petals, ethylene promoted the expression of *RhGLIP5*, whereas STS inhibited its expression. In addition, *RhIAA16*, a member of the AUX/IAA family in rose petal AZ, is involved in the positive regulation of the petal abscission process through an ethylene-independent pathway or upstream of the ethylene pathway (Gao et al., 2016). Interestingly, expression of a homologous family member, *RhIAA26*, distinguished other auxin-related genes from the antagonistic effect with ethylene, and the expression of *RhIAA26* was up-regulated under ethylene treatment, whereas STS had little effect on its expression. Moreover, the expression of *RhJAO2* (2-oxoglutarate-dependent dioxygenase) and *RhMGL* (methionine gamma-lyase) did not change under ethylene treatment but were up- or down-regulated under STS treatment. In conclusion, the expression of TFs, amino acid synthesis-related genes, cell membrane-related genes, and phytohormone-related genes were affected by STS in the AZ of rose petal. Further analyses were required to elucidate the molecular mechanisms of these genes in abscission process of rose petal.

The gene *RhMLP423* (pathogenesis-related protein) was selected among the 12 randomly screened DEGs. Its expression was down-regulated by STS and up-regulated by ethylene. In tobacco, *NtMLP423* regulates drought tolerance by increasing the ABA content under drought stress, and overexpression of *NtMLP423* reduces membrane damage and reactive oxygen species (ROS) accumulation (Liu et al., 2020). In animal cancer cells, STS promotes the accumulation of ROS (Ota et al., 2021). *RhMYB108* expression was down-regulated by STS treatment and up-regulated by ethylene. In cassava, the MYB transcription factor *MeMYB108* reduces leaf abscission under drought stress by inducing ROS scavenging (Wang et al., 2022). Whether ROS play a role that is affected by STS in the abscission of rose petals remains to be studied.

Polyamines (PAs) are cationic compounds that widely present in organisms and participate in fruit set (Alburquerque et al., 2006; Gomez-Jimenez et al., 2010). *PAO*, a gene associated with polyamine catabolism, is a stress-related gene. In present study, the expression of *RhPAO4* was down-regulated by STS and up-regulated by ethylene. Polyamines regulate ethylene biosynthesis in relation to nitric oxide (NO) and hydrogen peroxide (H_2_O_2_) during fruit abscission (Parra-Lobato and Gomez-Jimenez, 2011; Gil-Amado and Gomez-Jimenez, 2012). The gene *PAO4* is negatively correlated with salt stress and drought stress in tomato (Upadhyay et al., 2021). The expression of *ZmPAO2*, *3*, *4*, *5*, and *6* is increased in maize under drought stress (Pakdel et al., 2020). However, studies of the role of *PAO* genes in petal abscission of rose are presently lacking.

Ethylene response factors (AP2/ERFs) are associated with plant organ abscission (Yi et al., 2021). In the current study, we identified an ERF gene, *RhERF110*, that has not been reported previously in abscission-related studies. In cucumber, *CsERF110* is associated with sex determination and in Arabidopsis *AtERF110* regulates the timing of bolting (Zhu et al., 2013). We also screened an ethylene receptor gene, *RhETR2*, which is associated with abscission. The present qRT-PCR results showed that the expression of *RhERF110* and *RhETR2* was significantly down-regulated under STS treatment and significantly up-regulated in response to ethylene treatment. The functions of these genes in petal abscission of rose in response to STS treatment requires further investigation.

### 4.4 Integral effect of STS on DAMs and directly associated pathways, and genes

A combined analysis of metabolomic and transcriptomic KEGG enrichment results revealed a high degree of similarity between them. Meanwhile, STS exerted an effect on D-Erythrose 4-phosphate, an intermediate of the Pentose phosphate pathway, which is involved in the biosynthesis of various amino acids. And we found a differential gene *LOC112188495* (glyceraldehyde-3-phosphate dehydrogenase gene) in the Pentose phosphate pathway associated with D-Erythrose 4-phosphate biosynthesis (**Figure 8B**). Based on the expression pattern of the Pentose phosphate pathway and amino acid related DAMs, we found opposite trends in the effects of STS and ethylene on the contents of gluconolactone, D-Sedoheptulose 7-phosphate, D-Erythrose 4-phosphate, L-glutamic acid, protocatechuic acid, shikimic acid, L-Threonine, indole, and L-Tyrosine (**Figure 7**). Meanwhile, according to the classification results of amino acid- associated DEGs, four DEGs were found in group STS24h vs. MOCK24h, 66 DEGs of this class were found in group ETH24h vs. MOCK24h, and nine DEGs were differentially expressed in group S_E24h vs. MOCK24h. Thus, we hypothesized that ethylene had a dramatic effect on the transcription and synthesis of amino acids during the petal abscission of rose, both at the metabolite level and at the gene level, STS eliminated this effect by inhibiting ethylene synthesis.

We identified 1120 metabolites in the AZ of rose petals. Most DAMs in the AZ were annotated with five metabolite categories, comprising Global and overview maps, Amino acid metabolism, Biosynthesis of other secondary metabolites, Carbohydrate metabolism, and Metabolism of cofactors and vitamins (**Figure 2D**). Subsequently, 50 DAMs from these five categories were screened (**Figure 2E**). Among these DAMs, the contents of five DAMs were significantly affected by STS but did not change significantly under ethylene treatment: shikonin (naphthoquinones), jasmonic acid (phytohormones), gluconolactone (esters), stachyose (carbohydrates), and D-erythrose 4-phosphate (carbohydrates). A KEGG pathway enrichment analysis of the DAMs and DEGs indicated that 39 DEGs may be associated with these five metabolites (**Figure 8B**). Meanwhile, these 39 DEGs belonged to 10 KEGG pathways. Among them, Ubiquinone and other terpenoid-quinone biosynthesis and Vitamin B6 metabolism belongs to Metabolism of cofactors and vitamins; Galactose metabolism belongs to Carbohydrate metabolism; alpha-Linolenic acid metabolism belongs to Lipid metabolism; Plant hormone signal transduction belongs to Signal transduction; Pentose phosphate pathway belongs to Carbohydrate metabolism; Carbon metabolism and Biosynthesis of amino acids belongs to Global and overview maps; Carbon fixation in photosynthetic organisms belongs to Energy metabolism; Phenylalanine, tyrosine and tryptophan biosynthesis belongs to Amino acid metabolism. Among the six shikonin-related DEGs, the gene *LOC112192149* encoded a 4-coumarate coenzyme A ligase 6 (4CL6), which was up-regulated in expression under STS treatment and down-regulated under ethylene treatment. The role of 4CL is to catalyze the synthesis of 4-coumaric acid coenzyme A followed by the ultimate synthesis of *p*-hydroxybenzoic acid, a precursor for shikonin biosynthesis (Suttiyut et al., 2022). Although the reactions involved in this process are obscure, 4CL is a crucial enzyme for paclitaxel synthesis. The initial steps of jasmonate biosynthesis involve members of several gene families, including phospholipases (PLA/PLD), lipoxygenases (LOX), allene oxide synthase (AOS) and allene oxide cyclases (AOC) (Singh et al., 2022). In the present study, the content of jasmonic acid was up-regulated by STS and was associated with two DEGs (*LOC112196726* and *LOC112189737*). *LOC112196726* encodes a lipoxygenase 6 (LOX6) and its expression was down-regulated by STS but up-regulated by ethylene. An additional jasmonic acid-related gene, *LOC112189737*, encodes a jasmonate ZIM domain-containing protein which expression was promoted by STS and repressed by ethylene. In the Pentose phosphate pathway, D-erythrose 4-phosphate is generated from 3-phosphoglyceraldehyde, which in turn can be converted to 3-phosphoglyceric acid, and finally the interconversion of D-erythrose 4-phosphate and 3-phosphoglyceric acid occurs. Among the 27 DEGs associated with D-erythrose 4-phosphate, the expression of two glyceraldehyde-3-phosphate dehydrogenase genes (*LOC112188495* and *LOC112188936*) was up-regulated by STS and down-regulated by ethylene. In summary, five metabolites (shikonin, jasmonic acid, gluconolactone, stachyose, and D-erythrose 4-phosphate), and 39 DEGs were involved in the delay of petal abscission by STS. In addition, the genes 112192149 (4 coumaric acid-CoA ligase 6), 112196726 (lipoxygenase 6), 112189737 (jasmonic acid ZIM structural domain-containing protein 1), and two glyceraldehyde-3-phosphate dehydrogenase genes (112188495 and 112188936) were crucial factors related to the biosynthesis of shikonin, jasmonic acid, and D-erythrose 4-phosphate, so they probably may directly influence the changes in the three metabolic contents.

## 5 Conclusion

**Figure 9:**
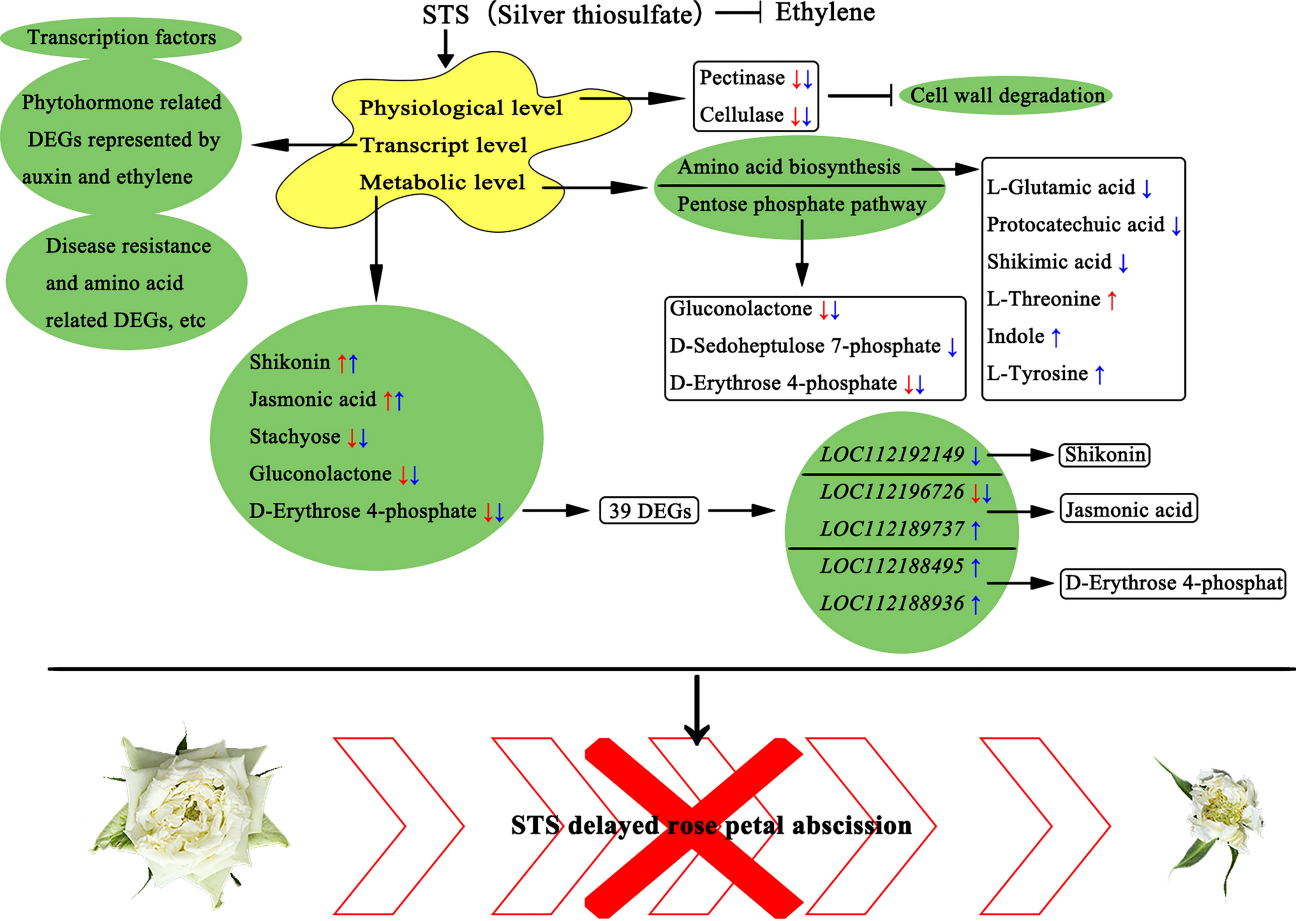
** **A proposed model of STS negatively influences petal abscission of rose. The red arrows indicated the up- or down- regulated in STS24h vs. MOCK24h; the blue arrows indicate the up- or down- regulated in STS24h vs. Eth24h. Scale bar, 5 cm.

Above all, we speculated a model by which STS negatively influences petal abscission of rose (**Figure 9**). STS significantly delayed the petal abscission in phenotype and reduced the activity of two enzymes (pectinase and cellulase) associated with cell wall degradation in physiological level. STS significantly affected the contents of five metabolites, namely shikonin, jasmonic acid, gluconolactone, stachyose, and D-erythrose 4-phosphate, and 39 DEGs associated with these five metabolites were screened, among them five DEGs (*LOC112192149*, *LOC112196726*, *LOC112189737*, *LOC112188495*, and *LOC112188936*) were probably directly associated with the biosynthesis of shikonin, jasmonic acid, and D-erythrose 4-phosphate. Meanwhile, STS had an effect on gluconolactone, D-Sedoheptulose 7-phosphate and D-Erythrose 4-phosphate in the pentose phosphate pathway and affected changes in various amino acid biosynthesis-related metabolites, including L-Glutamic acid, protocatechuic acid, shikimic acid, L-Threonine, indole, and L-Tyrosine. In addition, STS affected the expression changes of a large number of transcription factors, phytohormone related DEGs represented by auxin and ethylene, and DEGs related to disease resistance and amino acid biosynthesis.

## Data availability statement

The original contributions presented in the study are publicly available. This data can be found here: NCBI, PRJNA880356.

## Author contributions

JZ, HP, YZ, YH, ZQ, and CX conceived and designed the experiments. JZ, TD, and WW collected the rose accessions and participated in the material preparation. JZ, DS, and HF performed the experiments and did the formal analysis. JZ and HP analyzed the data and drafted the manuscript. All authors contributed to the article and approved the submitted version.

## Funding

This research was supported by National Nature Science Foundation of China to HP (Grant no. 31860572) and National Nature Science Foundation of China to CX (Grant no. 32060498).

## Acknowledgments

We sincerely thank the editors and reviewers for their contributions. We are grateful to the staff of Beijing Novogene Technology Co., Ltd (Beijing, China) for their support in metabolite determination and analysis and transcriptome detection and analysis.

## Conflict of interest

The authors declare that the research was conducted in the absence of any commercial or financial relationships that could be construed as a potential conflict of interest.

## Publisher’s note

All claims expressed in this article are solely those of the authors and do not necessarily represent those of their affiliated organizations, or those of the publisher, the editors and the reviewers. Any product that may be evaluated in this article, or claim that may be made by its manufacturer, is not guaranteed or endorsed by the publisher.
