# Supplementary material for: Metabolome and transcriptome integration reveals insights into the process of delayed petal abscission in rose by STS
Source: Front Plant Sci. 2022 Nov 15;13:1045270. doi: 10.3389/fpls.2022.1045270 (PMC9706100; doi:10.3389/fpls.2022.1045270)
Supplement: Supplementary file 1 [file DataSheet_1.zip › Supplementary Material Presentation/Supplementary Material.docx]

***Supplementary material***

**Supplementary Figures**

**
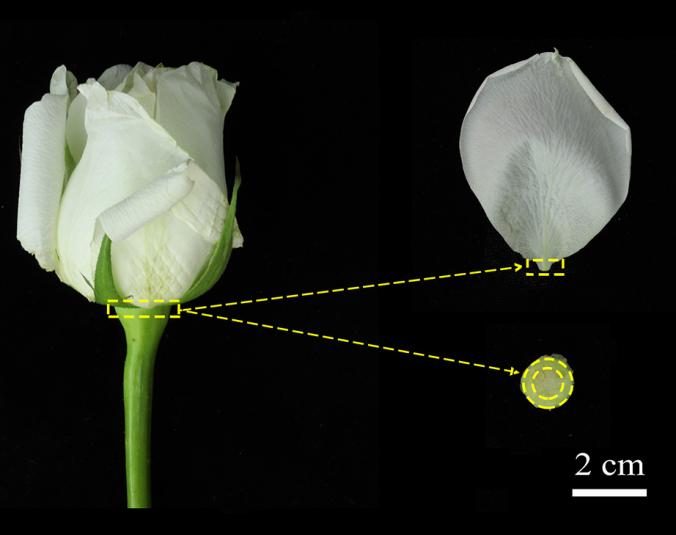
**

**Supplementary Figure 1.** Diagram of location for AZ samples. Scale bar 2 cm.

**
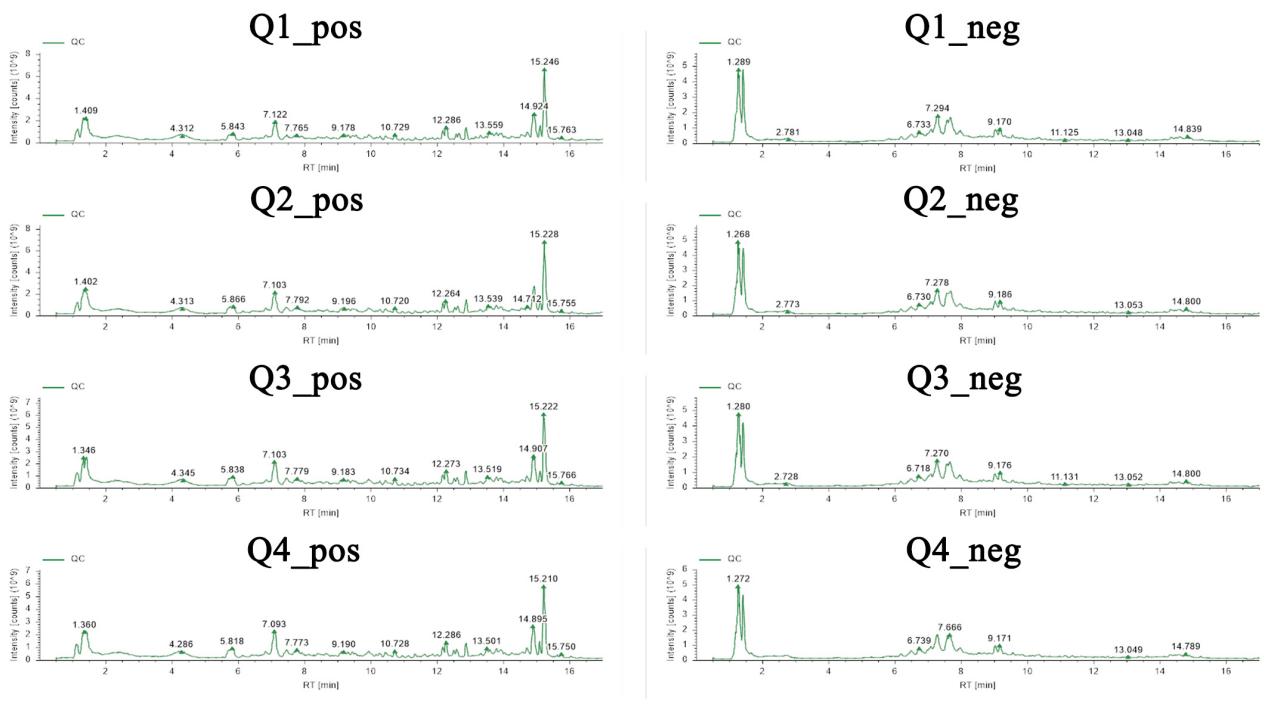
**

**Supplementary Figure 2.** Total Ion Chromatogram (TIC) of Mass Spectrometry Analysis. Pos indicated positive ion mode; neg indicated negative ion mode.

**
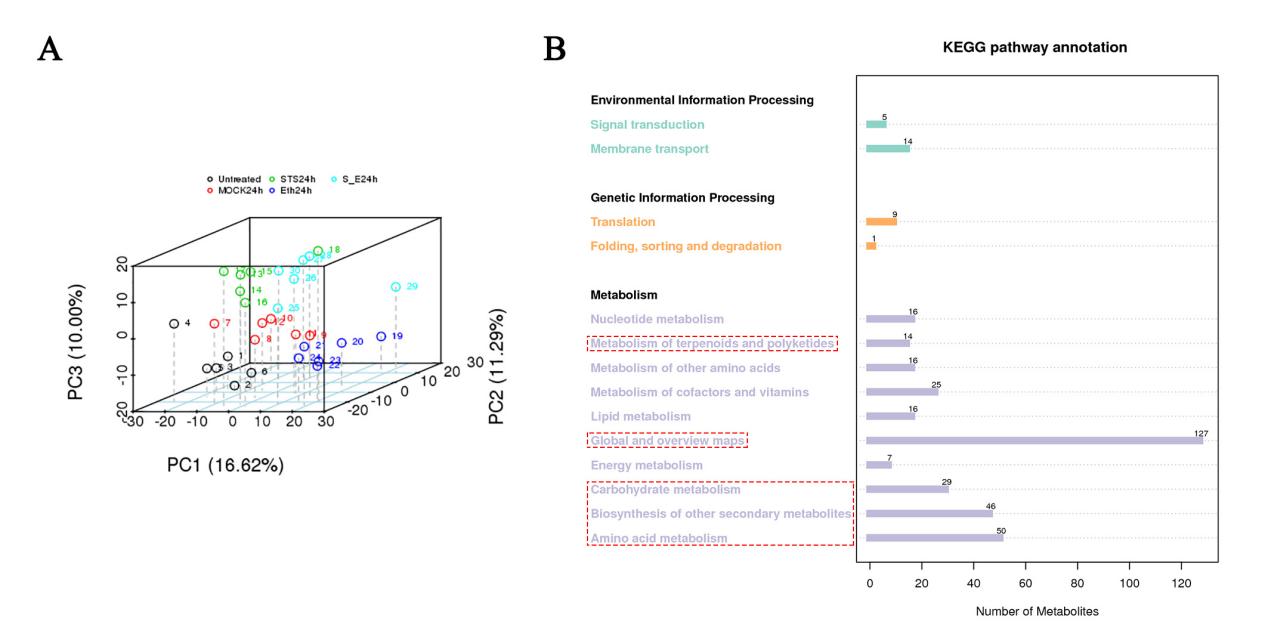
**

**Supplementary Figure 3.** PCA analysis and metabolite KEGG annotation of rose AZ under different treatments. **(A)** Principal component analysis (PCA) of metabolites from 30 samples. The samples were comprised of Untreated, MOCK24h, STS24h, ETH24h, and S_E24h, each sample with six biological replicates. **(B)** Annotation of 351 metabolites by KEGG pathway database. Different colors represented different level 1 of KO_Pathway. The content of the red dashed box was the KEGG category with a high number of metabolites.


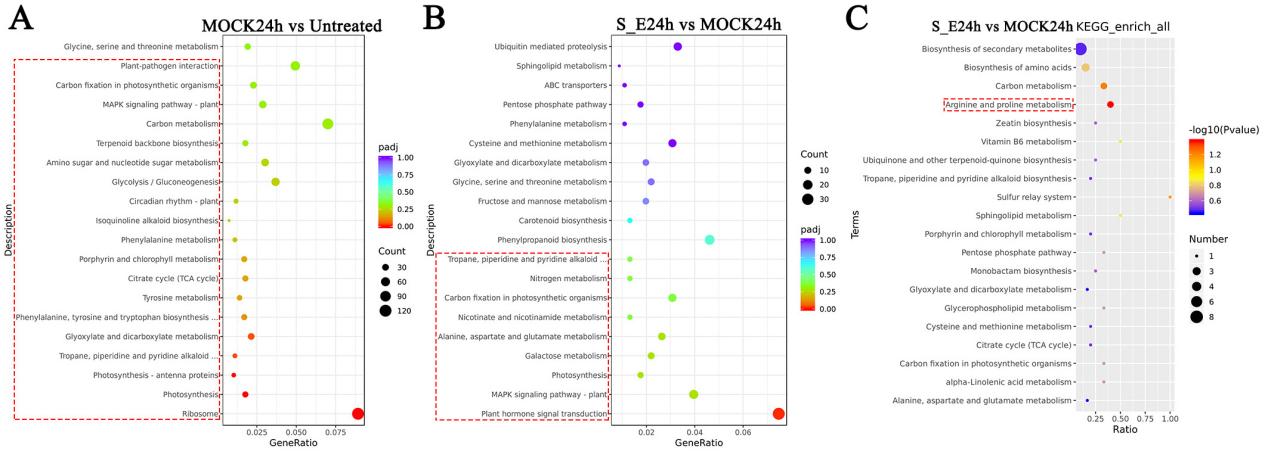


**Supplementary Figure 4.** KEGG enrichment analysis in MOCK24h vs Untreated and S_E24h vs MOCK24h. **(A)** and **(B)** KEGG enrichment analysis of DEGs. **(C)** KEGG enrichment analysis of DAMs in S_E24h vs MOCK24h. The content in the red dashed box was the significantly enriched (p-value < 0.05) KEGG pathway.

**
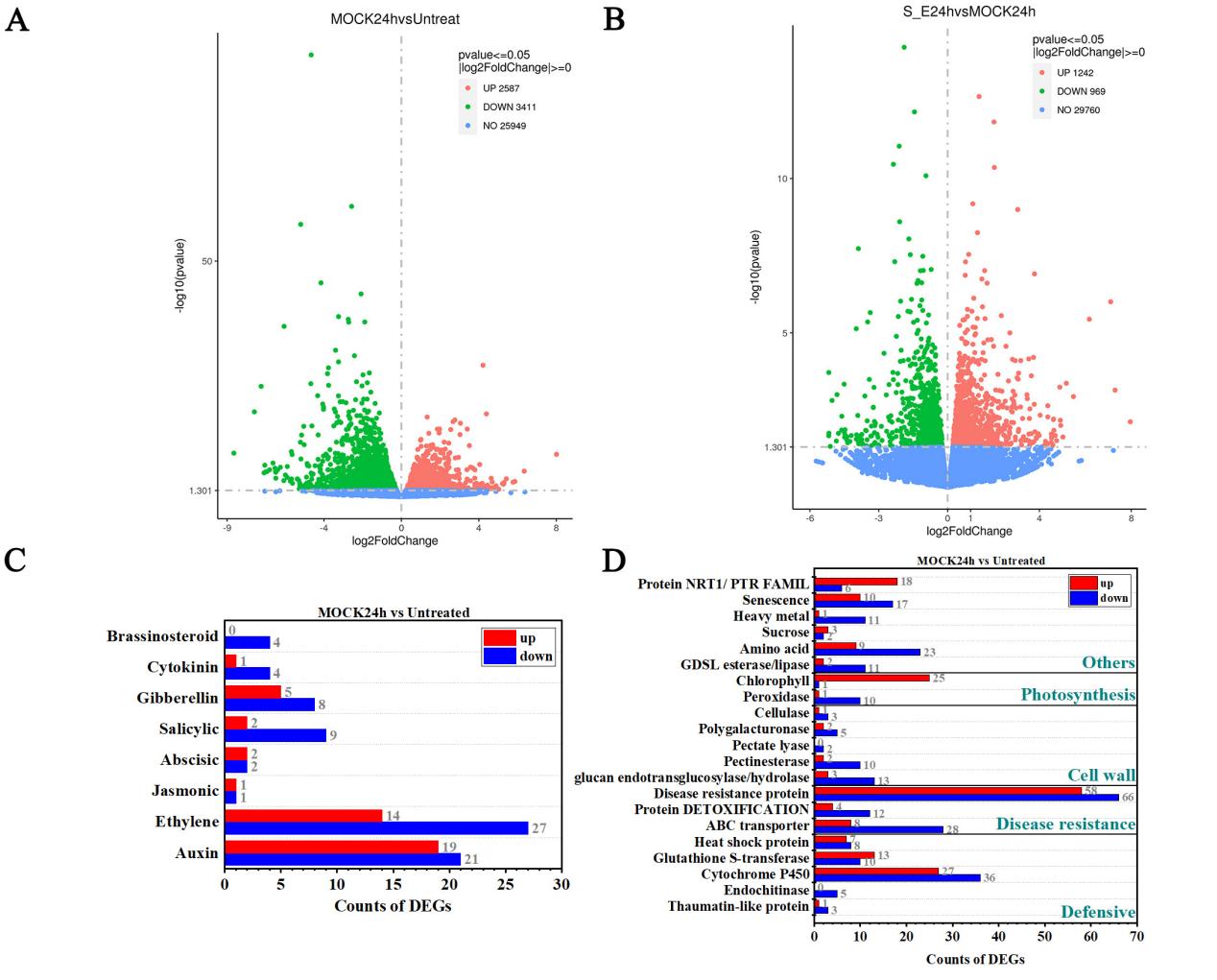
**

**Supplementary Figure 5.** Volcano plots and classification of DEGs in MOCK24h vs Untreated and S_E24h vs MOCK24h. **(A)** and **(B)** Volcano maps of DEGs. **(C)** and **(D)** Classification of hormone-related DEGs. **(E)** and **(F)** Classification of different function-related DEGs.
